# Comparative transcriptomics among peach, almond and their interspecific F1 hybrid reveal key common and species-specific regulatory pathways involved in fruit development

**DOI:** 10.1186/s12870-026-08863-6

**Published:** 2026-05-12

**Authors:** Ioannis Kandylas, Beatriz E. García-Gómez, Naveen Kalluri, Konstantinos Alexiou, José Tomás Matus, David Navarro-Payá, Pere Arús, Iban Eduardo

**Affiliations:** 1 Centre for Research in Agricultural Genomics (CRAG) CSIC-IRTA-UAB-UB, Campus UAB, Edifici CRAG, Cerdanyola del Vallès (Bellaterra), Barcelona, 08193 Spain; 2 IRTA, Campus UAB, Edifici CRAG, Cerdanyola del Vallès (Bellaterra), Barcelona, 08193 Spain; 3 Institute for Integrative Systems Biology (I2SYSBIO, UV-CSIC), Paterna, Valencia, 46980 Spain

**Keywords:** *Prunus*, RNA-Seq, Mesocarp development, Fruit ripening, Allele-specific expression

## Abstract

**Background:**

Peach (*Prunus persica*) and almond *(P. dulcis*) are closely related species within the *Prunus* genus that exhibit strikingly different fruit characteristics, particularly in mesocarp expansion and ripening behaviour. To investigate the biological processes driving these differences, we performed a comprehensive transcriptomic analysis of fruit development in the peach cultivar ‘Earlygold’, the almond cultivar ‘Texas’, and their interspecific F1 hybrid. Fruit samples were collected at three developmental stages that are key in the different ripening behaviour of peach and almond: initial phase of rapid growth (T1), cell expansion and lignification (T2), and ripening (T3).

**Results:**

Global transcriptome profiling revealed almost identical expression patterns irrespective of the reference genome used for the RNA-seq analysis. We found 4,241, 3,862 and 2,922 DEGs between T1 and T2 in ‘Earlygold’, ‘Texas’ and F1 hybrid respectively, with most specific changes (55%, 76.6% and 51.3%) occurring during the first half of fruit development. Between T2 and T3, peach-type fruits continued active transcriptional regulation (2,665 DEGs in ‘Earlygold’, 2,199 in F1 hybrid), whereas almond showed limited late-stage changes (1,032 DEGs), reflecting its non-ripening phenotype. Enrichment analysis showed conserved cell division and photosynthesis-related genes enriched at T1 at both species. Peach displayed unique enrichment in pathways related to auxin signaling, DNA replication, and cyanogenic compound metabolism whereas almond for abscisic acid- and ethylene-related stress pathways. Allele-specific expression (ASE) analysis in the F1 hybrid revealed 79, 99 and 119 peach-biased ASE genes, and 27, 51 and 77 almond-biased ASE genes at T1, T2 and T3, respectively.

**Conclusions:**

These findings reveal that peach and almond share conserved early developmental programs but diverge markedly from mid-development. Our data highlight auxin signaling, DNA replication, and ethylene-mediated ripening as central processes driving these developmental differences. The limited number of ASE genes and their parental bias patterns further illuminate cis-regulatory divergence between both species. This study provides new insights into the genetic regulation of fruit development in *Prunus* species and demonstrates a robust pipeline for cross-species transcriptomic analysis.

**Supplementary Information:**

The online version contains supplementary material available at 10.1186/s12870-026-08863-6.

## Introduction

Peach [*Prunus persica* L. (Batsch)] and almond [*Prunus dulcis* (Miller) DA Webb.] are drupe-type fruits belonging to the *Prunus* genus and the *Amygdalus* subgenus. They exhibit distinct fruit typologies and are cultivated for different purposes. Peaches are grown for their fleshy mesocarp, whereas almonds are grown for their non-bitter edible kernel. These two closely related species are both diploid (2n = 2x = 16) and possess relatively small and highly syntenic genomes [1] of approximately 227 Mb for peach [2] and 254 Mb for almond [3]. Their main differences at the genomic level are attributed to the presence and distribution of transposable elements [4]. In the past, interspecific crosses between the almond cultivar ‘Texas’ and the peach cultivar ‘Earlygold’ enabled the development of an F2 population, named TxE, which played a key role in the construction of the *Prunus* reference genetic map [5]. Following the marker assisted introgression strategy (MAI) [6], which combines successive backcrossing and whole-genome marker assisted selection, researchers developed the first collection of introgression lines (ILs) for a perennial woody species, using peach as the recurrent parent [7, 8]. In these TxE cross-derived populations, two major genes distinguishing peach and almond fruit have been mapped. *Alf*, which controls ripening ability, and *Jui*, which regulates fruit juiciness [7].

Over the past century, peach fruit development has been extensively studied at physiological, molecular, and transcriptomic levels. Peach fruit growth follows a double sigmoid curve comprising four distinct stages (S1 to S4). In S1, rapid fruit growth occurs primarily through active cell division. During S2, growth slows as endocarp lignification takes place. Growth resumes in S3, continuing until reaching its final size in S4, when growth stabilizes and ripening begins [9–11]. Fruit enlargement in S3 and S4 is mainly driven by cell expansion, involving cell wall loosening, cell separation, intercellular space formation, and endoreduplication [12, 13]. In contrast, almond fruit development is generally divided in three stages. Initially, the mesocarp and endocarp expand while the embryo and cotyledons remain liquid (up to 80 days after flowering (DAF)). In the second stage (up to 110 DAF), the embryo and endosperm become visible, and the endocarp begins to harden and brown. During the final stage, fruit growth ceases, the cotyledons fully occupy the seed coat, the endocarp completely hardens, and the drying non-ripening mesocarp and exocarp split to expose the endocarp [14, 15].

Rosaceae fruits are classified as either climacteric, characterized by increased respiration and ethylene production during ripening (e.g., apples, pears, peaches, plums, apricots), or non-climacteric, where ripening is typically regulated by abscisic acid (ABA) (e.g., strawberries, *Rubus spp*., some “suppressed climacteric” plum cultivars) [16, 17]. Almond fruit, however, with its non-fleshy, non-ripening mesocarp, does not conform to either category. In climacteric fruits, ethylene and auxin–ethylene interactions regulate ripening [18, 19]. Recent studies have identified NAC transcription factors as key regulators of climacteric ripening in species lacking whole-genome duplication, such as peach, papaya, and melon [20]. The NAC-NOR gene in tomato is a well-characterized regulator of ripening [21], and its homolog CmNAC-NOR regulates melon ripening [22]. In peach, maturity date trait has been fine-mapped to a genomic region of chromosome G4, where two NAC transcription factors, *PpNAC1* and *PpNAC5*, have been identified as key regulators of ripening [23, 24].

Comparative transcriptomic strategies are influenced by the degree of species relatedness, involving approaches such as mapping transcriptomes to single or combined reference genomes and analyzing expression patterns of orthologous genes as it has been done in several species [25–29] including peach and almond [30]. Peach transcriptomic studies have extensively explored fruit development and [11, 31–34], light and stress responses [35], and postharvest life [36] between others. Almond RNA-seq studies mainly investigated flower bud dormancy [37], self-incompatibility [38], fruit drop [39], and kernel bitterness [40]. Additionally, RNA-seq enables the detection of single-nucleotide polymorphisms (SNPs) within coding sequences (CDS), facilitating allele-specific expression (ASE) analysis in heterozygous or hybrid species [41]. ASE studies in maize hybrids have identified genes overlapping with heterosis-related quantitative trait loci (QTLs), revealing promoter variations influencing yield [42, 43]. In apple hybrids (*Malus* x *domestica*), ASE analysis uncovered transposable element insertions affecting flower color [44]. Similarly, in lotus (*Nelumbo nucifera*), cis-regulatory divergence was shown to influence rhizome enlargement and phenotypic differentiation between ecotypes [45].

This study investigates transcriptomic changes during mesocarp development in peach cultivar ‘Earlygold’, almond cultivar ‘Texas’, and their interspecific F1 hybrid. By comparing gene expression profiles at critical developmental stages, we aimed to identify transcriptional changes and main biological processes underlying peach and almond developmental and mesocarp ripening differences. RNA-seq analyses involved alignment to (i) species-specific genomes and (ii) solely the peach genome, after identifying corresponding genes and genome-specific transcripts. Additionally, ASE in the F1 hybrid was analyzed to assess parental allele expression patterns during mesocarp development.

## Methods

### Plant material and fruit development monitoring

In this study, we used the peach commercial cultivar ‘Earlygold’, the almond commercial cultivar ‘Texas’, and their interspecific F1 hybrid developed and maintained at IRTA experimental station in Torre Marimon, Caldes de Montbuí, Spain. In 2018, fruit development was monitored from anthesis to ripening, periodically measuring the diameter of five fruits per genotype. Based on these growth profiles, fruit samples were collected in 2019 at three key stages: stage 1 (T1), prior to exponential growth when all fruits were the same size; stage 2 (T2), midpoint between T1 and maturity; and stage 3 (T3), at maturity indicated by skin color change and decreased firmness. In almond, maturity corresponded to mesocarp splitting. Fruits were peeled, the flesh was diced, frozen immediately in liquid nitrogen, and stored at -80 °C until RNA extraction. Three biological replicates per sample were analyzed.

### RNA extraction and mRNA sequencing

Samples were ground into powder, and total RNA was extracted using PowerPlant RNA Isolation Kit^®^ (Qiagen, Germany), followed by DNase treatment with a DNAse On-Spin Column DNase I Kit (Qiagen). RNA purification and concentration were achieved using an UltraClean Plant RNA Isolation Kit (Qiagen). RNA quantity and purity were assessed using a NanoDrop^®^ One Spectrophotometer (Thermo Scientific), normalized to 0.5 µg (50 ng/µL). Integrity was verified on a 2100 Bioanalyzer (Agilent), and qualified samples underwent library preparation. RNA-seq libraries were generated from mRNA-enriched total RNA and sequenced via paired-end sequencing on the HiSeq2000 platform (Illumina).

### Bioinformatic analyses

#### Sequencing libraries, mapping to reference genomes

Sequencing libraries, mapping, and quality control Raw FASTQ reads were filtered for quality using Trim Galore v0.6.1, removing reads with Phred scores < 30 and Illumina adapters. High-quality reads were mapped to peach (‘Lovell v2.1’) [2] and almond (‘Texas v.3 – Phase 1’ reference genomes using HISAT2 v2.1.0 [46] with default settings. Alignment and mapping quality were evaluated with SAMStat v1.5.1 [47] and SAMtools v1.9 [48], retaining only reads with MAPQ ≥ 30 (Table S1). Sorted and indexed BAM files underwent further analyses. FastQC v0.11.8 [49] and MultiQC v1.9 [50] generated quality reports.

#### Differential gene expression analysis

Read count matrices were generated using featureCounts (Rsubread R package v3.8) [51], configured for paired-end reads without chimeric fragments and gene ID annotations. Differentially expressed genes (DEGs) were identified with DESeq2 v1.30.1 [52], applying a preliminary filter (minimum 10 counts in three samples), a Benjamini-Hochberg (BH) method derived adjusted p-value ≤ 0.05, and a log2 fold change (logFC) threshold > |1|. For within-species comparisons, respective reference genomes were used, while the peach reference genome was used for between-species analyses. The earliest timepoint served as the reference for within-species analyses, and ‘Earlygold’ served as reference for between-species comparisons.

#### Gene set enrichment analysis

Gene set enrichment analysis (GSEA) for Kyoto Encyclopedia of Genes and Genomes (KEGG) pathways and Gene Ontology (GO) terms was performed using *clusterProfiler* package in R, applying the *gseKEGG* and *GSEA* functions with default settings and adjusted p-value thresholds of 0.1 and 0.05, respectively. GSEA detects coordinated expression patterns within ranked gene lists, capturing subtle pathway enrichments beyond DEGs alone [53]. Before almond KEGG analysis, gene IDs were converted from Texas v.3 to v.2 [3, 4]. GO enrichment for biological processes (BP) was performed using the *enricher* function on up- and downregulated genes for each genotype. Databases for GO terms and KEGG IDs were obtained from the Ensembl Plant BioMart database (https://plants.ensembl.org). PFAM domain lists were sourced from the Genome Database for Rosaceae (GDR) [54] and processed using a custom Python script.

#### Gene correspondence analysis

To transfer gene annotations between genomes, we used *liftoff* with default parameters without looking for additional copies [55]. After lifting the annotations, we performed a coordinate-based overlap analysis at the gene, exon and coding sequence (CDS) levels to determine the degree of correspondence between annotated features in the query and target genomes. This analysis yielded gene-level overlap percentages for each pair of matching features, following the methodology described in [56] and implemented using the AEGIS package (https://github.com/Tomsbiolab/aegis). A valid gene correspondence list was defined by retaining only those pairs with minimum reciprocal overlaps of 50% at the gene level, 30% at the exon level, and 10% at the coding sequence (CDS) level, resulting in an overlap score ≥ 6. Final one-to-one gene correspondences were defined by removing one-to-many and many-to-one gene matches. Euler diagrams were created with the eulerr R package [57].

#### Allele-Specific Expression (ASE) analysis

Candidate ASE sites were identified from SNP lists obtained via variant calling of whole-genome resequencing data from ‘Earlygold’, ‘Texas’, and the F1 hybrid mapped to the peach genome as described by [58].Germline variants were identified using GATK v4.1.7 and filtered according to GATK’s Best Practices, excluding multiallelic variants [59]. The informative SNPs retained were heterozygous in the F1 hybrid (0/1) and homozygous in ‘Earlygold’ (0/0) and ‘Texas’ (1/1) within CDS regions. Allele-specific counts from RNA-seq data mapped to the Lovell v2.1 genome were obtained using GATK’s ASEReadCounter [59]. Genes with at least two SNPs per CDS and average allele frequencies above 95%, with a standard deviation below 2 across replicates, were considered ASE.

### Quantitative real-time PCR

cDNA was synthesized from 1 µg total RNA using the PrimeScript RT-PCR Kit (Takara, Japan). Quantitative real-time PCR (qPCR) reactions contained 50 ng cDNA in a 10 µL reaction with 1X SyberGreen Mix, using the Roche LightCycler 480 II system. Cycling conditions included denaturation at 95 °C for 5 min, followed by 40 cycles at 95 °C for 10 s, 60 °C for 15 s, and 72 °C for 20 s. Housekeeping genes Actin (Prupe.6G163400-TexasF1_G21887) and CDKA (Prupe.2G084600-TexasF1_G17410) were selected for stable expression across samples. Primers were designed using Primer-BLAST (NCBI), targeted exon-exon junctions and were verified manually for specificity to both ‘Earlygold’ and ‘Texas’ transcriptomes.

## Results

### Description of fruit development in peach, almond and their F1 interspecific hybrid

**Fig. 1:**
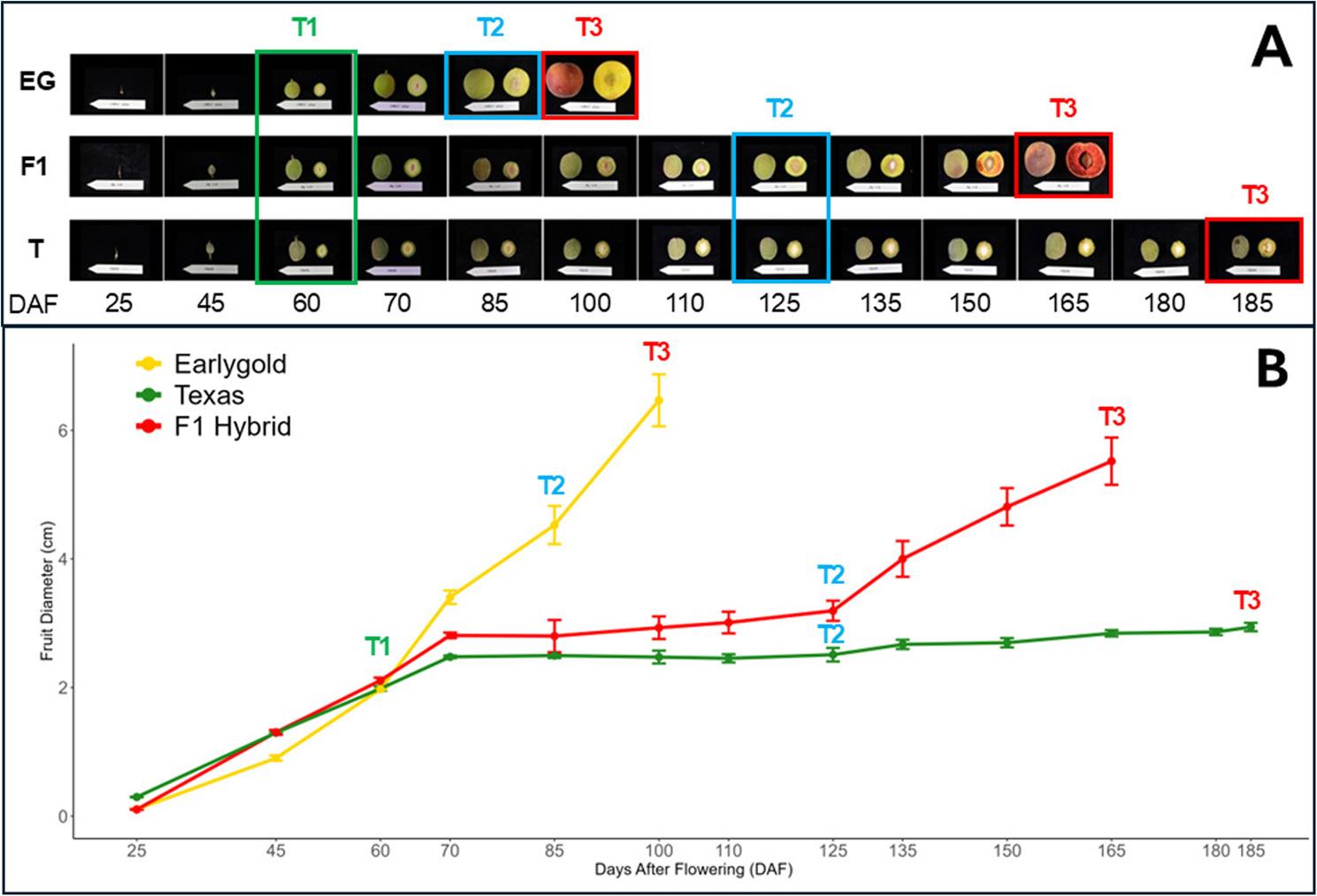
Fruit development of ‘Earlygold’ (EG) peach, ‘Texas’ (T) almond and their interspecific F1 hybrid (F1). **A** Pictures of fruits during fruit development. **B** Fruit growth curves. Samples used in the transcriptomic study are highlighted in boxes for T1 (same size stage), T2 (middle point of development) and T3 (maturity date/mesocarp splitting)

We measured the fruit diameter of peach ‘Earlygold’, almond ‘Texas’, and their interspecific F1 hybrid from 25 DAF to maturity date in peach and mesocarp splitting in almond (Fig. 1). Until 60 DAF, the three genotypes followed the same linear growth pattern. After this period, each fruit followed its own growth pattern. ‘Earlygold’ continued growing exponentially, reaching maturity five weeks later (100 DAF). ‘Texas’ ceased growing two weeks after T1 (70 DAF), maintaining a constant fruit diameter until the mesocarp split (185 DAF). The F1 interspecific hybrid, a late-maturing peach type, displayed a typical double-sigmoid growth pattern, as previously described in peach [11].

### Transcriptomic analyses of peach almond and the F1 hybrid during fruit mesocarp development

Based on the distinct growth behaviors of the three genotypes, we selected three stages of fruit development for sample collection in our transcriptomic study. The first timepoint (T1) was when all three genotypes had reached the same fruit size (approximately 2 cm) (60 DAF). The second timepoint (T2) was a middle point between T1 and the maturity date of each fruit (85 DAF for peach and 125 DAF for F1 hybrid and almond). The final time point (T3) corresponded to the maturity date for the peach-type fruits, 100 DAF for ‘Earlygold’ and 165 DAF for F1 hybrid and mesocarp splitting in almond, 185 DAF.

**Fig. 2:**
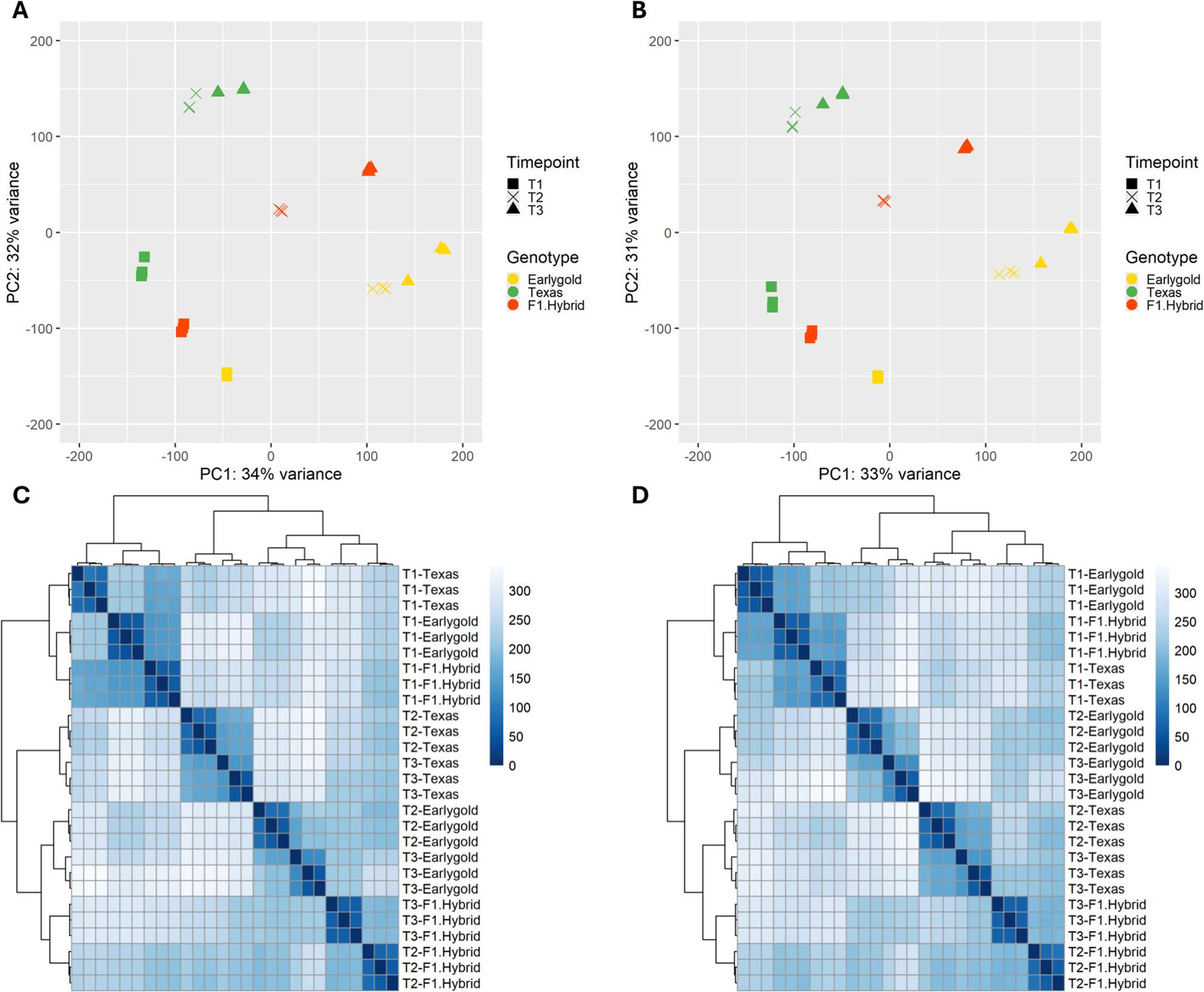
Exploratory analysis and visualization of r-log transformed expression data. The filtered and normalized data used for exploratory analysis were obtained from reads mapped to Lovell v2.1 **A**, **C** and Texas v.3 **B**, **D** reference genomes. Principal component analysis (PCA) and a sample-to-sample Euclidean distance matrix heatmap were generated to assess the overall similarity between samples

According to the PCA plots (Fig. 2), both PC1 (31–32% of the explained variation, depending on the reference genome used) and PC2 (33–34%) presented similar patterns across all the samples. PCAs revealed that the gene expression patterns were very similar to those of both the Lovell v2.1 and Texas v.3 reference genome analyses. At T1, the three genotypes were closer to each other, indicating that the expression profiles of all three fruit mesocarps were more similar at T1 than at later developmental stages. At T2 and T3, the genotypes were more separated, with T2 samples being closer to T3 samples than to T1 samples.

The robust RNA-seq dataset enabled DEA within genotypes at consecutive timepoints and between genotypes at the same timepoints. As the F1 hybrid at T2 and T3 clustered closer to ‘Earlygold’ or ‘Texas’ when using the peach or almond reference genomes (Fig. 2C–D), we used F1 samples only for within-genotype DEA and for ASE analysis. Euclidean distance matrices and PCAs using only ‘Earlygold’ and ‘Texas’ confirmed a consistent data structure across reference genomes (Fig. S1).

#### DEA and enrichment analysis during peach, almond and F1 hybrid mesocarp development

**Fig. 3:**
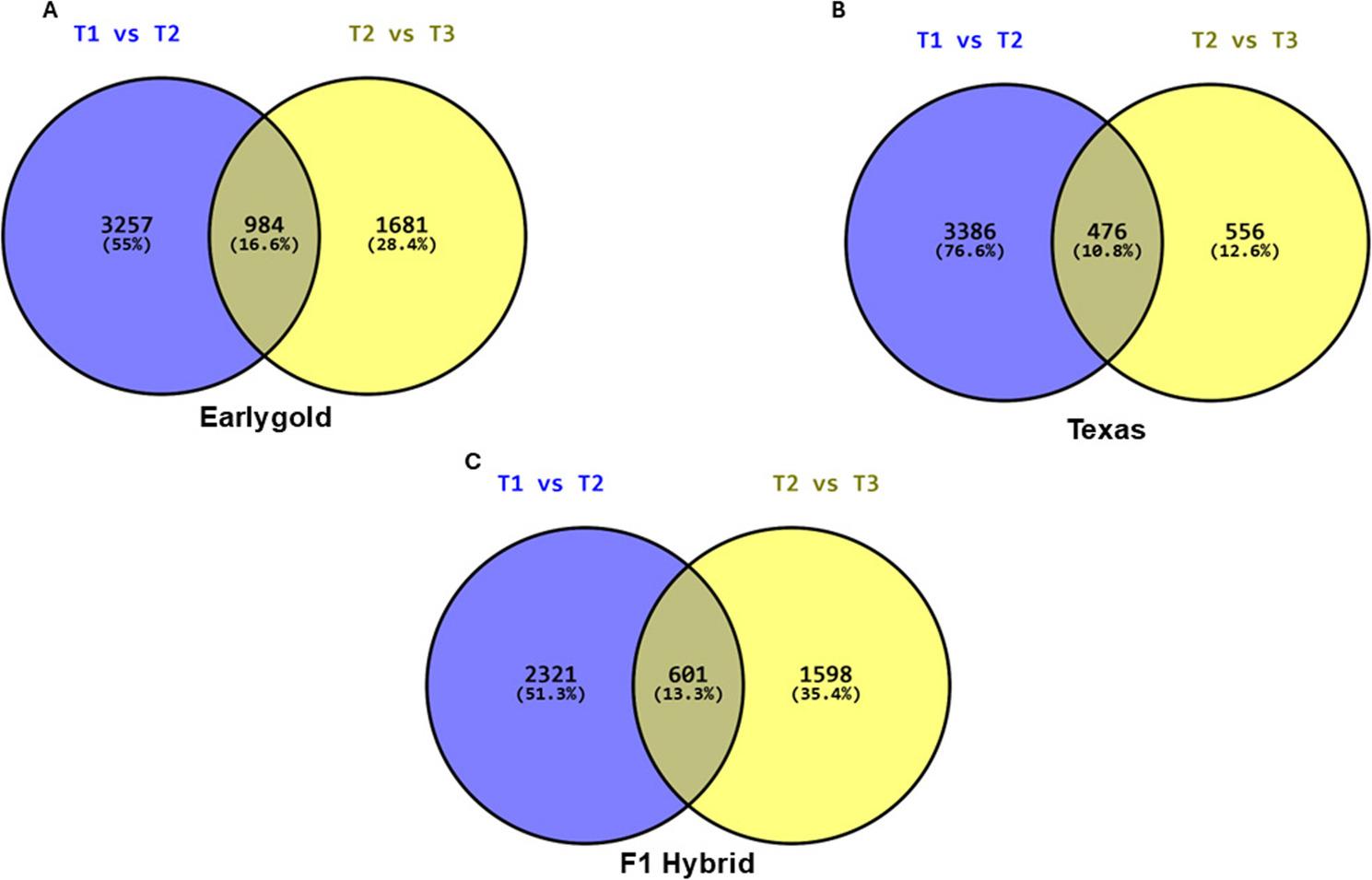
Venn diagrams obtained from DEG analyses between consecutive timepoints of peach ‘Earlygold’ (left), almond ‘Texas’ (right) and their F1 hybrid (middle) fruit development

We first performed DEA within each genotype at consecutive timepoints (Table S2-S7). ‘Earlygold’ presented the greatest number of DEGs between T1 and T2 (4,241 DEGs), followed by ‘Texas’ (3,862 DEGs) and the F1 hybrid (2,922 DEGs). Comparisons between T2 and T3 in ‘Earlygold’ revealed 2,665 DEGs, followed by the F1 hybrid with 2,199 DEGs and ‘Texas’ with 1,032 DEGs. The Venn diagram (Fig. 3) shows the total number of DEGs, including those specific to pairwise comparisons and those common between them. In all three fruits, the majority of DEGs were specific to T1 vs. T2, representing 55% of all DEGs in ‘Earlygold’, 51.3% in the F1 hybrid and 76.6% in ‘Texas’. The specific DEGs at T2 vs. T3 accounted for 28.4% in ‘Earlygold’, 35.4% in the F1 hybrid, and 12.6% in ‘Texas’. The common DEGs between T1 vs. T2 and T2 vs. T3 pairwise comparisons accounted for 16.6%, 13.3% and 10.8% of all DEGs during fruit development in ‘Earlygold’ and F1 hybrid and ‘Texas’, respectively. These findings indicate that ‘Earlygold’ and the F1 hybrid undergo active transcriptomic changes between T1 and T2 and between T2 and T3, whereas ‘Texas’ shows mainly transcriptomic shifts between T1 and T2, which are limited between T2 and T3 despite being sampled 60 days apart.

**Fig. 4:**
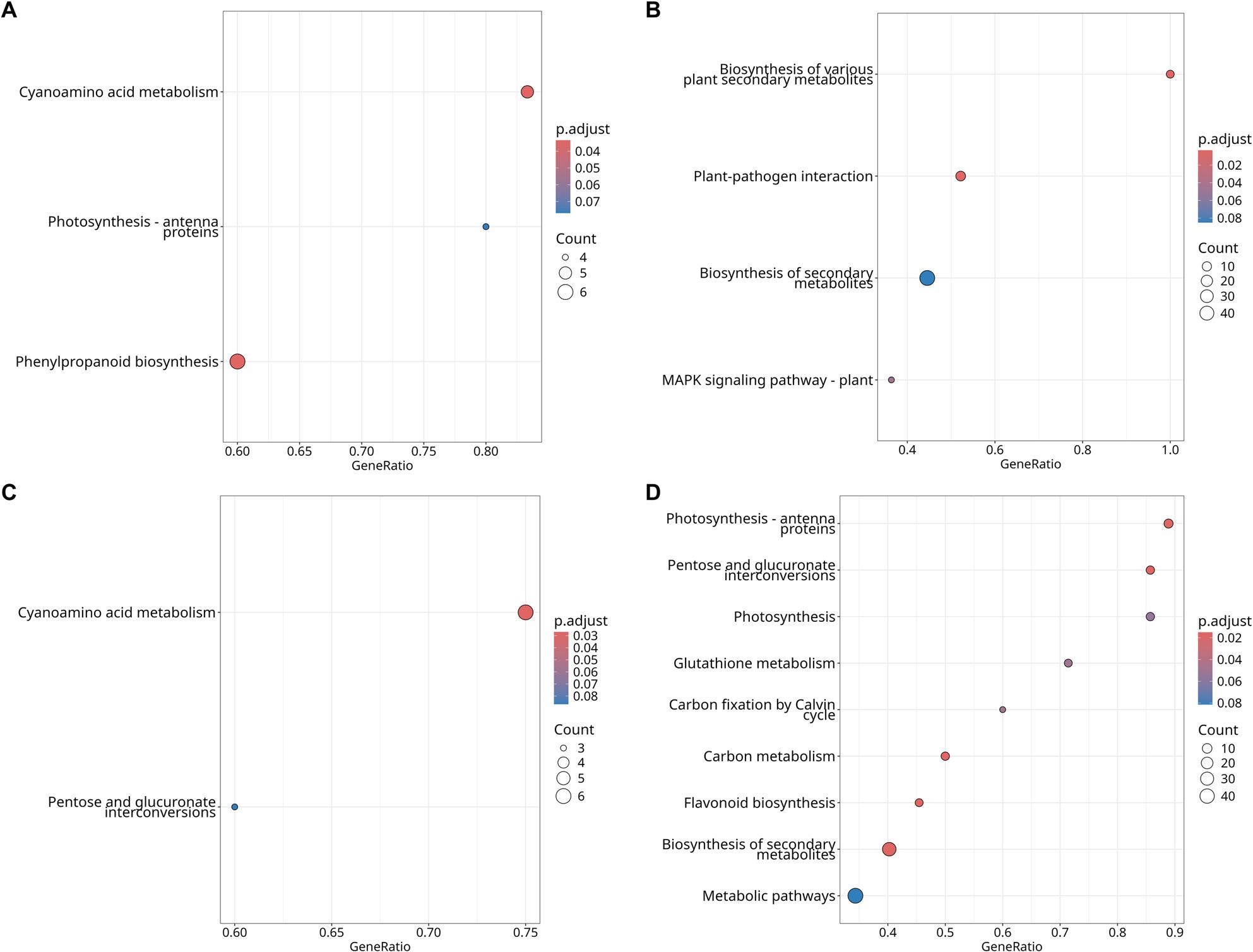
Gene set– enrichment analysis dot plots with the most enriched KEGG pathways for the DEGs in A) ‘Earlygold’ T1 vs T2, B) Texas’ T1 vs T2, C) F1 hybrid T1 vs T2, and D) F1 hybrid T2 vs T3

Gene set enrichment analysis (GSEA) for KEGG pathway enrichment analysis highlighted the most enriched pathways between consecutive timepoints in ‘Earlygold’, ‘Texas’ and F1 hybrid for the ranked (ordered by logFC) DEG gene lists. GSEA identifies whether a gene set is significantly enriched at the top or bottom of a ranked list, revealing coordinated activation or repression of pathways that may not be detected through standard differential expression alone [53]. For ‘Earlygold’, downregulated genes between T1 and T2 were enriched in *phenylpropanoid pathway* (*p*-adjusted = 9.6 e-04), with four peroxidases and two shikimate O-hydroxycinnamoyltransferases; *cyano aminoacid metabolism* (*p*-adjusted = 5.9 e-04) with five β-glucosidases; one mandelonitrile lyase (MDL); and *photosynthesis-antenna proteins* (*p*-adjusted = 0.0038), with four light-harvesting chlorophyll a/b (Lhc a/b)-binding proteins (Fig. 4A, Table S2). In ‘Texas’, upregulated genes between T1 and T2 (Fig. 4B) were associated with *plant‒pathogen interactions* (*p*-adjusted = 0.0039), including four WRKY-TFs, two disease resistance proteins, and the *MAPK-signaling pathway-plant* (*p*-adjusted = 0.079), including three WRKY-TFs and the aminocyclopropane-1-carboxylate synthase (*PdACS1*). Enrichment was detected for the *biosynthesis of various secondary metabolites* (*p*-adjusted = 0.004), including among others a polyphenol oxidase (PPO). At T2 vs. T3, both in ‘Earlygold’ and ‘Texas’ DEGs were not mapped to any pathways (Table S3, S5).

In the F1 hybrid, downregulated genes between T1 and T2 (Fig. 4C) were enriched in the *cyanoamino acid metabolism* pathway (*p*-adjusted = 0.045), including a MDL gene, and three β-glucosidases, common with ‘Earlygold’ at this stage. Conversely, upregulated genes in this interval were enriched in the *pentose and glucuronate interconversion* pathway, featuring a sorbitol dehydrogenase (*PpSDH*), an endopolygalacturonase (*PpPG22*) and an exopolygalacturonase. Between T2 and T3, the downregulated genes were enriched in photosynthesis-related processes, including *antenna proteins* (*p*-adjusted = 0.013), and *photosynthesis* (*p*-adjusted = 0.013) genes common with ‘Earlygold’ at T1. Between T2 and T3 (Fig. 4D), upregulated genes were enriched in *flavonoid biosynthesis* (*p*-adjusted = 0.016), including a chalcone synthase (*PpCHS*), a chalcone isomerase (*PpCHI*), a flavonol synthase (*PpFLS*), and a leucoanthocyanidin dioxygenase (*PpLDOX*). Additionally, enrichment in *pentose and glucuronate interconversions* (*p*-adjusted = 0.016) persisted, with continued upregulation of *PpSDH* and *PpPG22* reported earlier, together with a pectin methylesterase inhibitor (PMEI), aldo-keto reductase (*PpAKR*), and two pectate lyases (*PpPL*). *Glutathione metabolism* (*p*-adjusted = 0.016) was also enriched, with four glutathione transferases (*PpGST*), together with broader categories of *secondary metabolite biosynthesis* (*p*-adjusted = 0.016) and *metabolic pathways* (*p*-adjusted = 0.061).

To complement the gene set KEGG analysis, gene set enrichment analysis for Gene Ontology (GSEA-GO) terms was performed for T1 vs. T2 and T2 vs. T3 within the genotype. The most enriched GO terms for the pairwise comparisons between consecutive timepoints in ‘Earlygold’, ‘Texas’ and the F1 hybrid are listed in Tables S2-S7, and their logFC trends are shown in Fig. S2 and Fig. S3.

#### Enrichment of biological processes associated with up- and downregulated genes during peach and almond mesocarp development

**Fig. 5:**
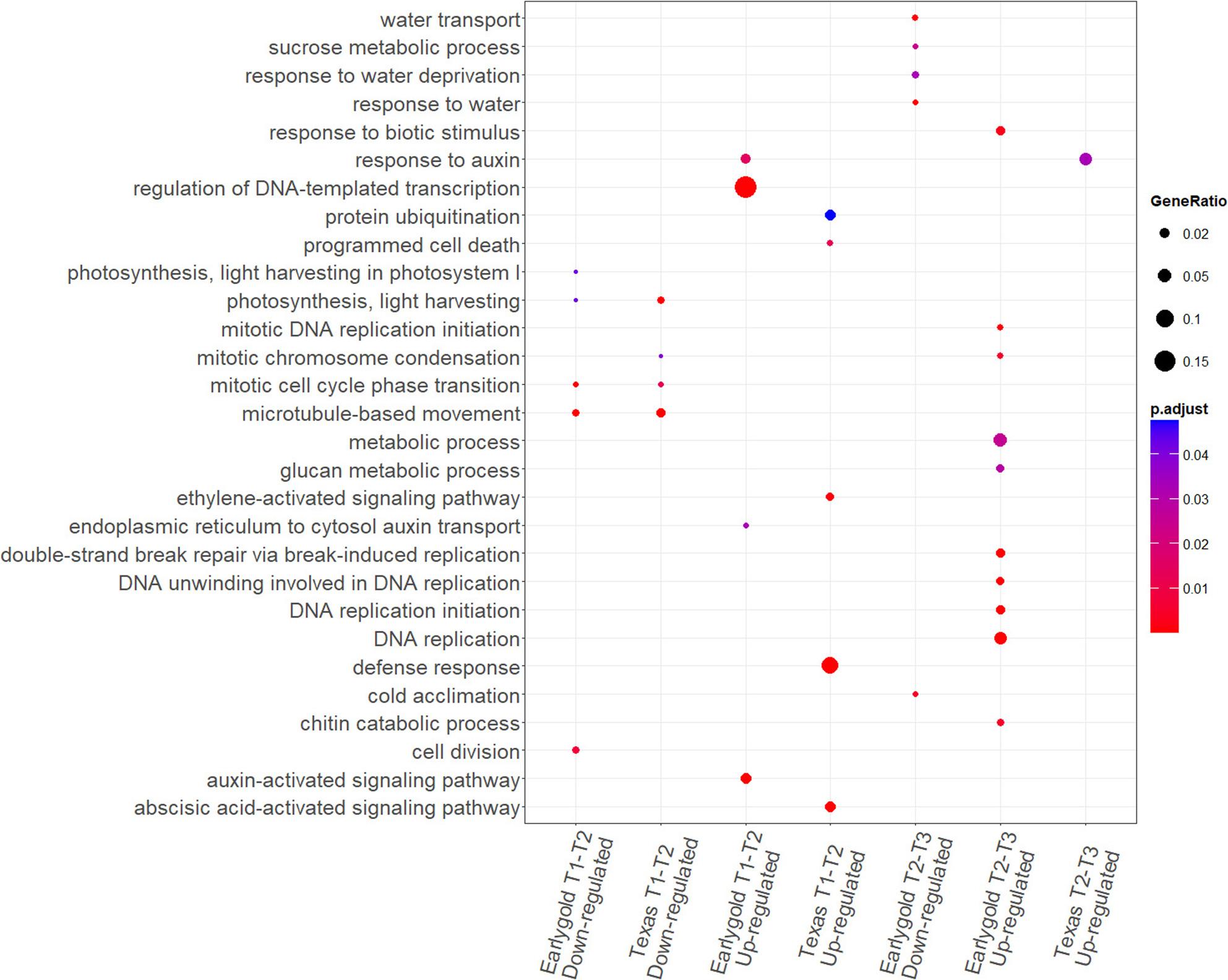
Gene Ontology biological process enrichment dot plots for the up- and downregulated genes at each timepoint within the ‘Earlygold’ fruits and within the ‘Texas’ fruits.

To further elucidate biological processes during peach and almond fruit development, we analyzed ‘Earlygold’ and ‘Texas’ separately. We performed GO enrichment for up- and downregulated genes between T1 and T2 and between T2 and T3, and PFAM domain identification of enriched genes to determine the gene families involved (Fig. 5, Table S8).

In ‘Earlygold’, there were 4,241 DEGs between T1 and T2, with 1,609 upregulated and 2,632 downregulated genes. Enrichment analysis highlighted several BP terms that were downregulated, including cell division-related terms, such as “microtubule-based movement”, “mitotic cell cycle”, and “cell division”, including twenty-four genes with kinesin motor domains and thirteen cyclins and photosynthesis–related terms (“photosynthesis, light harvesting/in photosystem I”,) with nine light-harvesting chlorophyll a/b-binding proteins (Lhc a/b). The upregulated BPs included auxin-related genes, such as “auxin activated signaling pathway”, “response to auxin” “endoplasmic reticulum to cytosol auxin transport”, eighteen small auxin-up RNA (SAUR) family genes, fifteen auxin/indole-3-acetic acid (AUX/IAA) genes and six PIN-Auxin efflux carrier family genes (PIN) “regulation of DNA-templated transcription”, eighteen AP2/ethylene responsive factor (ERF) genes, thirteen MYB genes, twelve NAC-domain factor genes, twelve zinc finger C2H2-type genes, eight basic helix-loop-helix (bHLH) genes, five zinc finger B-box type genes, five B3-domain TF genes, four WRKY genes and five AUX/IAA genes, four WRKY genes and three basic-leucine zipper (bZIP) genes.

In ‘Earlygold’ a total of 2,665 DEGs were found between T2 and T3, with 1,092 upregulated and 1,573 downregulated genes. The downregulated terms were “water transport”, “response to water”,“cold acclimation” and “response to water deprivation” including ten plasma membrane intrinsic protein (PIP)-aquaporins and five dehydrins (DHNs). Additionally, “sucrose metabolic process” was slightly enriched, including three sucrose phosphate synthases (SPSs) and three sucrose synthases (SuSy). The upregulated genes at T3 were enriched in DNA replication processes, such as “DNA replication”, “double stranded break repair via break-induced replication”, “DNA replication initiation”, “DNA duplex unwinding/involved in replication” and “mitotic DNA replication initiation”, including six minichromosome maintenance proteins (MCMs), six DNA replication GINS complex proteins and two ribonucleotide reductases (RNRs), two cell division control proteins (CDCs) and two helicases. Finally, we found enrichment for “metabolic process”, “glucan metabolic process” and “chitin catabolic process” which consisted mainly of glycosyl- hydrolase members with at least six xyloglucan endotransglucosylases (XTH), five polygalacturonases (PG), three β-galactosidases, three phosphofructokinases, one mananyl-oligosaccharide glucosidase (GS1) and one mannan endo β-mannosidase (MAN).

In almond, pairwise comparisons revealed a total of 3,862 DEGs for T1 vs. T2, with 1,968 upregulated and 1,894 downregulated genes. Enrichment of the downregulated genes revealed, like those in ‘Earlygold’, cell division-related processes, with ten microtubule-associated proteins, five kinesins and five cyclins and “photosynthesis, light harvesting” including ten Lhc a/b-binding genes.

The upregulated genes in T1 vs. T2, were associated with “abscisic acid-activated signaling pathway”, with twenty major latex-like proteins (MLPs) and pathogenesis-related (PR) members, “defense response” and “programmed cell death”, with twenty-three MLPs, twenty-one TIR domain proteins, eight NLR resistance proteins, six mild resistance (MLO) proteins, five MAC/perforin proteins and few other disease resistance genes. Interestingly, we identified an “ethylene-activated signaling pathway” with eleven AP2/ERF factors. A slight enrichment was detected for “protein ubiquitination, with twenty-one zinc-finger superfamily members.

In T2 vs. T3, a total of 1032 DEGs were found, with 422 upregulated and 610 downregulated genes. With respect to the GO enrichment analysis, we only found a slight “response to auxin” enrichment for the upregulated genes, including five SAUR family genes, whereas the downregulated genes were not enriched with any term.

### Differential expression between peach and almond fruits during fruit mesocarp development

**Fig. 6:**
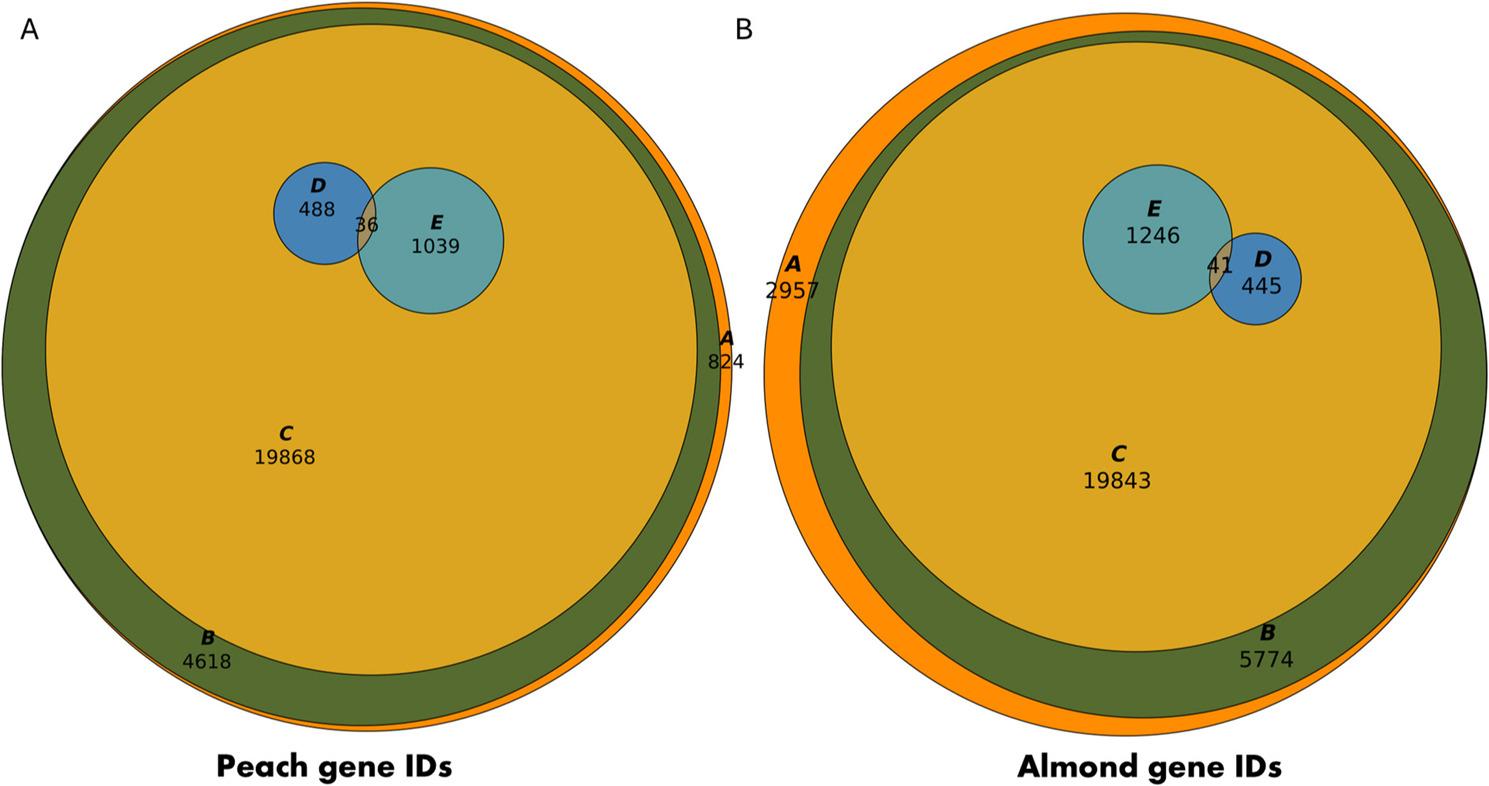
Venn diagrams representing gene annotation relationships between peach and almond genomes. **A** Mapping of peach gene models (Lovell v2.1) onto the almond reference genome (Texas v.3). **B** Mapping of almond gene models onto the peach reference genome. In each diagram, proportional segments indicate the following categories: A) Genes that could not be lifted (genome-specific genes), B) Lifted genes without a valid correspondence, **C** One-to-one gene correspondences, **D** One-to-many (1:*n*) correspondences, and **E** Many-to-one (*n*:1, with *n* > 1) correspondences. The sum of the numbers represents all the gene models annotated in each respective genome

To assess genome similarity between peach and almond and support transcriptomic comparisons, we first mapped all annotated genes of one genome to the other using *liftoff* in both directions [55]. Orthologous and corresponding genes between peach and almond were identified using the AEGIS package (Fig. 6). Most genes were successfully lifted between genomes, with 824 peach genes (3.06%) and 2,957 almond genes (9.75%) not lifted, suggesting species-specific content (Fig. 6A). A further 4,618 (17.19%) peach and 5,774 (19.05%) almond genes did not reach the correspondence threshold (overlap score < 6; Fig. 6B) and were excluded. Using an overlap score ≥ 6, we retained 21,431 (79.75%) peach and 21,575 (71.19%) almond genes with valid correspondences (Fig. 6C–E).

To perform DEA between ‘Earlygold’ and ‘Texas’ across the three stages, we used one-to-one gene correspondences, focusing on almond genes with clear orthologs in the peach genome, resulting in 19,843 gene pairs (Table S9), of which 16,130 were expressed. Mean expression values were highly correlated (*r* = 0.978) between ‘Earlygold’ and ‘Texas’ regardless of the reference genome (Fig. S4). Given the higher BUSCO completeness scores of the peach genome for both genome assembly and annotation (Fig. S5), we selected the peach reference genome as the standard reference for DEA in this study.

DEA identified 1,721 DEGs at T1, 5,197 at T2, and 5,066 at T3 (Table S10-S12). The lowest number of DEGs at T1 increased notably at T2 and T3, indicating enhanced transcriptomic divergence associated with ripening progression. Among the 100 genes with highest expression variance, 43 were peach-specific, mainly expressed at T2 and T3, and 41 were almond-specific, similarly expressed at later stages, while 16 were common to both species at T1 (Fig. S6). As expected, key ripening-associated genes highly expressed in peach were also detected among the DEGs. These included *PpPG21* (Prupe.4G261900), *PpPG22* (Prupe.4G262200) and *PpACS1* (Prupe.2G176900) were included, with the corresponding almond *PdACS1* ortholog (TexasF1_8820) and the corresponding ortholog of *PpPG21* (TexasF1_G16883) showing residual expression in ‘Texas’ at T2 and T3, respectively. Consistent with the upregulated genes found earlier in the ‘Earlygold’ T1 vs. T2 comparison, several auxin-related genes were also found among the ripening marker genes. Notably, two AUX/IAA members, *PpIAA17* (Prupe.7G247500) and *PpIAA5* (Prupe.8G232200), and one *PpGH3*.1 member (Prupe.8G137900), showed strong induction at the onset till then end of ripening (T2 and T3) in peach and were not expressed in almond. *PpIAA17* displayed log₂ fold changes (logFC) of 15.07 and 10.12 at T2 and T3, respectively, while *PpIAA5* showed logFC values of 14.76 and 12.71 at the same stages, relative to ‘Texas’.

**Fig. 7:**
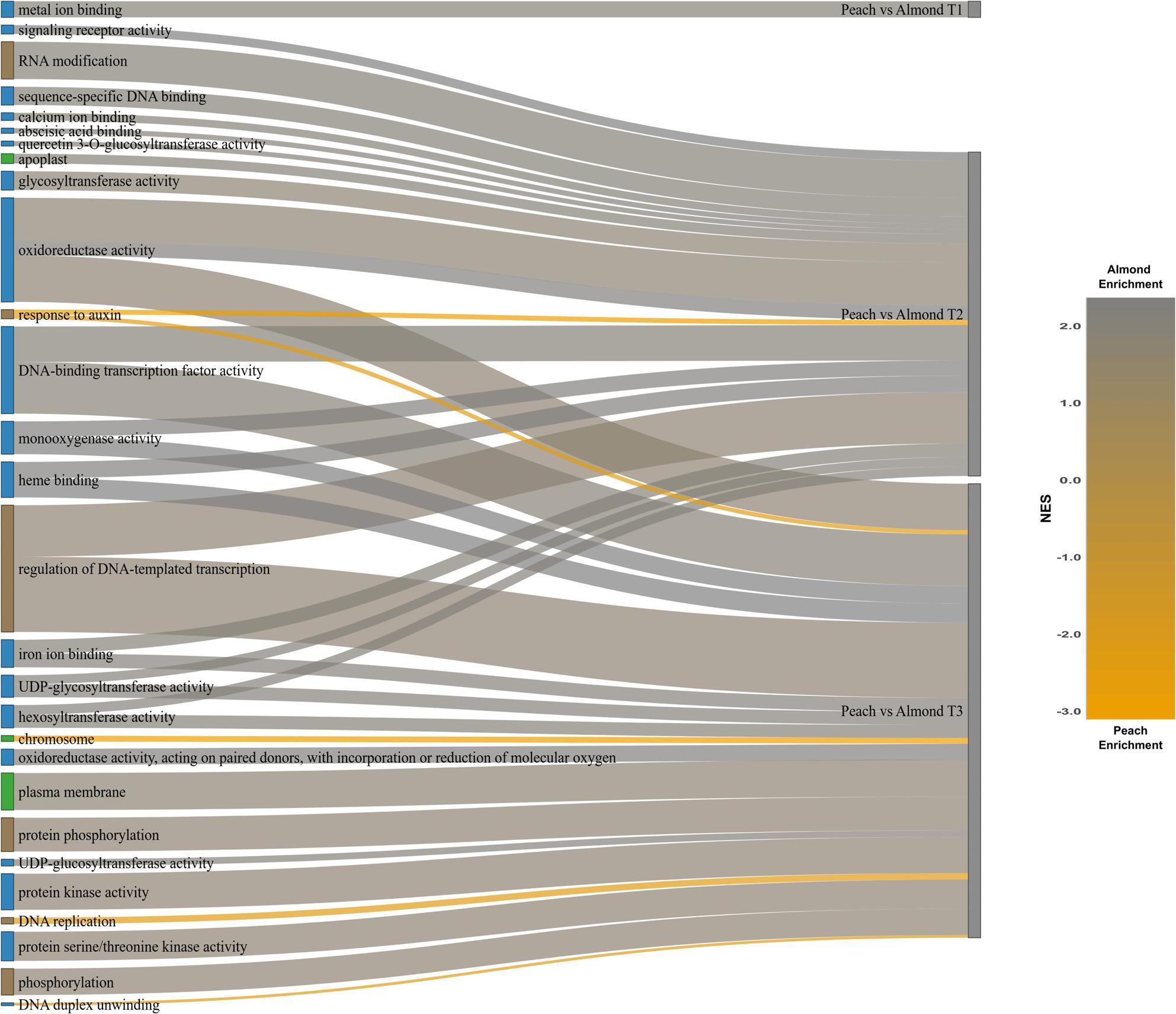
Sankey plot representing the top GO terms for the gene set enrichment analysis in the three pairwise comparisons between peach ‘Earlygold’ and ‘Texas’. Descriptions of the GO terms (MF: blue, BP: brown and CC: green) are represented at the left and linked with the corresponding pairwise comparisons at the right. The width of the links represents the number of genes enriched. Normalized enrichment scores (NES) are shown on the color scale, with positive values indicating almond-upregulated pathways and negative values indicating peach-upregulated pathways. Only the top 20 enriched GO terms with a q value ≤ 0.01 are presented

GSEA revealed key enriched GO terms across comparisons (Tables S10–S12, Fig. S7), with the most enriched shown in Fig. 7. The normalized enrichment score (NES) reflected the degree to which a gene set was overrepresented at the top or bottom of a ranked gene list, with positive values indicating enrichment in almond and negative values indicating enrichment in peach. At T1, “metal ion binding” was specifically enriched in almond, involving predominantly cytochrome P450 (CYP450) and terpene synthases. At T2, almond enriched genes included “RNA modification” and “apoplast”, highlighted by pentatricopeptide repeat (PPR) genes, XTHs, UDP-glucosyl transferases, MLA, and PR genes commonly involved in “signaling receptor activity” and “abscisic acid binding”. Conversely, almond T2 downregulated genes (i.e., upregulated in peach) were uniquely enriched in “response to auxin” to one of the most strongly enriched terms at all developmental stages. At T3, “plasma membrane”, “protein phosphorylation” and “protein kinase activity” were prominently enriched in almond, involving protein kinases, NB-LRRs, and wall-associated receptor kinase (WAK) genes. The highest almond-enriched processes, common to T2 and T3 were “oxidoreductase activity”, “monooxygenase activity” and “heme binding”, driven by CYP450s, peroxidases, and lycopene cyclases and “UDP-glycosyltransferase activity” represented by (galactosyl)transferases. DNA replication- related terms (including “chromosome”, “DNA-duplex unwinding”) were highly enriched in peach involving mainly MCM family members, and together with “response to auxin” represented the key peach-specific processes governing fruit development at T3. Notably, “response to auxin” was enriched at both T2 and T3, including numerous SAUR genes, with twelve overlapping with the earlier peach-specific development comparison in Earlygold T1 vs. T2 (Fig. S8A). To sum up, through DEA and GSEA between peach and almond, auxin- and DNA replication- related processes governed the main differences since the middle developmental stage.

“Regulation of DNA-templated transcription” prominently featured transcription factor families such as AP2/ERF, WRKY, bHLH, MYB, B3, and NAC. We further examined these genes because they might be related to fruit ripening, and we found that no genes were common specifically between ‘Earlygold T1 vs T2’ and ‘Earlygold T2 vs Texas T2’ (Fig. S8 B). Nevertheless, at T3 we identified sixty-seven upregulated genes that were common specifically between ‘Earlygold T1 vs T2’ and ‘Earlygold T3 vs Texas T3’. Among those, we identified one NAC TF that is known to control the maturity date of peach [60]. Surprisingly, the NAC5 ortholog (Prupe.4G186800-TexasF1_G15837) exhibited higher expression in ‘Texas’ than in ‘Earlygold’ at T2 and T3 (logFC values of 2.31 and 5.98 respectively). Regarding the other major candidate gene for peach fruit ripening, NAC1 (Prupe.4G187100-TexasF1_G15838) was expressed higher in Earlygold compared to Texas at T1 and T2 (logFC 2.44 and 3.4 respectively), indicating differential regulation of ripening-associated transcription factors between peach and almond.

We further examined almond-specific genes to investigate potential associations with the almond non-ripening phenotype. Among the 2,957 almond-specific genes identified (Fig. 6), 141 and 43 genes were differentially expressed between ‘Texas T1 vs. T2’ and ‘Texas T2 vs. T3’, respectively. At T1 vs. T2, “polysaccharide binding” was enriched, involving six Wall-associated receptor kinase (WAK) genes. At T2 vs. T3, enriched terms included “receptor kinase activity”, with FERONIA receptor-like kinases, “terpene synthase activity”, including an alpha-pinene, alpha-phellandrene synthase and costunolide synthases and “secondary metabolism” peroxidases and CYP450 genes (Table S13).

### F1 hybrid Allele**-**Specific Expression (ASE)

To determine whether there was a bias in parental allele expression during fruit development in the F1 interspecific hybrid, we conducted an ASE analysis, examining the transcriptomic data at each timepoint separately.

We examined ASE in the F1 interspecific hybrid during fruit development, using 2,225,586 informative SNPs. Of 22,118 genes retained after filtering, 14,688 were expressed at T1, 13,906 at T2, and 13,206 at T3. Using a stringent threshold (parental allele expression > 95%), we identified more peach-ASE than almond-ASE genes: 79 peach and 27 almond at T1, 99 peach and 51 almond at T2, and 119 peach and 77 almond at T3 (Table S14). Most ASE genes showed intermediate expression (71.46%) between parental values, while 22.56% showed positive transgression and 5.98% negative transgression. GO enrichment analysis highlighted “peroxidase activity” for peach-ASE at T1 and “secondary metabolic process” for almond-ASE at T1 and T2 (Table S15).

At T3, as almond-ASE genes we identified three pectin methyl esterase inhibitor (PMEI) genes enriched in “enzyme inhibitor activity” with one of them (Prupe.2G279700) showing a peach-ASE at T1. Regarding peach-ASE genes at T3 these were one lipoxygenase (*PpLOX3;* Prupe.4G047800), and “carotene catabolic process” with a carotenoid cleavage dioxygenase (*PpCCD4;* Prupe.1G255500), and an ABA biosynthetic gene, one nine-cis-epoxycarotenoid dioxygenase (*PpNCED3;* Prupe.4G150100) (Table S15). All genes with ASE were validated by visualizing the RNA-seq data in IGV. Notably, the ripening-related gene *PpIAA5*, shown to be highly induced in peach since T2 (Fig. S6), showed peach-ASE at T2 and T3 following an intermediate expression. Lastly, the PMEI demonstrating dual ASE behavior (peach ASE at T1, almond ASE at T3) showed also differences at a splicing site level while *PpNCED3* exhibited peach-ASE at T3 despite expression in both parents. A 14 bp deletion at an ERF binding site in the almond *PpNCED3* promoter might explain the peach-bias (Fig. S9).

### Validation of RNA-seq by qPCR

**Fig. 8:**
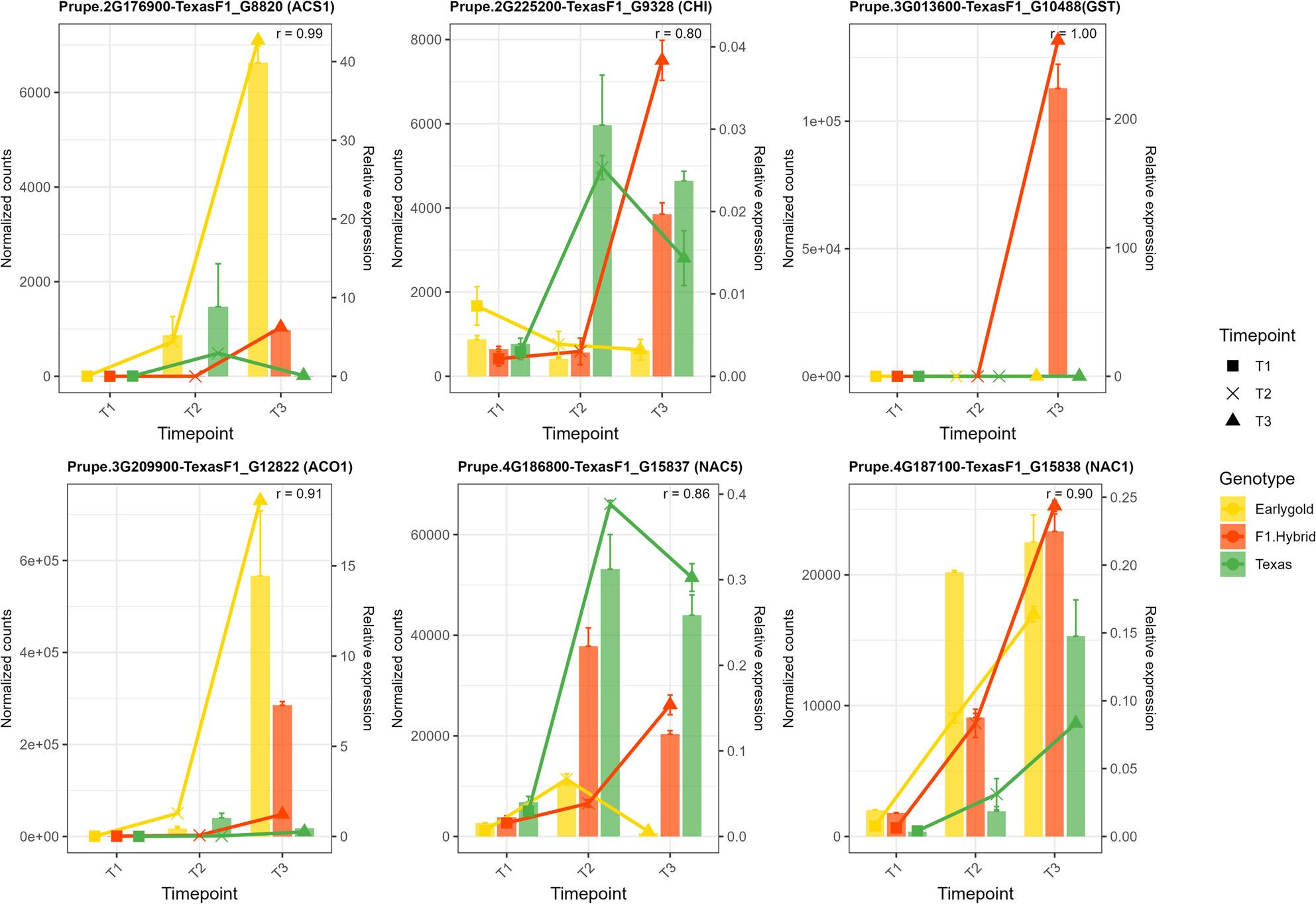
Gene expression plots for validation of RNA-seq normalized counts (axis y-left) with relative expression determined via qPCR (axis y-right). Bar plots showing the normalized (DESeq normalization) counts of each genotype using peach reference genome. Solid lines show the relative expression (RE) transformed data (RE/100) of each genotype compared with the expression of ‘Earlygold T1’. The error bars represent the standard deviation of the mean (*n*=3). Pearson correlation (*r*) in each plot shows the correlation between RNA-seq and qPCR data

To validate the RNA-seq expression profiles, we measured the relative expression of several genes in our samples via qPCR. We selected two *PpNAC* ripening regulators (*PpNAC1* and *PpNAC5*), the ethylene structural genes *PpACS1* and *PpACO1*, and the flavonoid pathway *PpCHI* and anthocyanin transporter *PpGST* genes enriched in the F1 hybrid at T3 (Fig. 4D). All of them presented a high correlation (*r* > 0.75) between RNA-seq and qPCR gene expression, validating the results obtained with the RNA-seq data (Fig. 8). Primers are listed in Table S16.

## Discussion

In this study, we investigated transcriptomic changes and regulatory mechanisms underlying mesocarp development in peach, almond and their interspecific F1 hybrid across three distinct fruit developmental stages. Through KEGG and GO enrichment analyses, we identified key biological processes regulated in each species during early and late fruit development. Additionally, we examined allele-specific expression (ASE) patterns in the F1 hybrid to gain insights into regulatory divergence between both species.

### Differences in the fruit growth phases, between almond, early-ripening peaches and late-ripening peaches

The fruit growth trajectories of ‘Earlygold’ and the F1 hybrid, both producing peach-type fruits, differed markedly from that of ‘Texas’, which produce almond-type fruits. Up to 60 DAF, all genotypes followed similar linear growth. Thereafter, ‘Earlygold’ underwent exponential growth until maturity, while ‘Texas’ ceased growth shortly after T1, maintaining a stable fruit size. The F1 hybrid displayed the typical double-sigmoid pattern observed in peach. In contrast, ‘Earlygold’, an early-ripening cultivar, showed a shortened S2 phase, consistent with previous findings [11]. In almond, only the S1 and S2 stages were observed, as previously reported [14], suggesting that key species-specific differences arise in the S2–S3 transition. Transcriptome profiling supported this divergence: at T1, all genotypes shared similar transcriptomic profiles, but from T2 onward, the almond transcriptome remained relatively stable, while peach and F1 hybrid displayed major transcriptomic shifts (Figs. 2 and 3), mirroring their developmental patterns.

### Common biological processes between peach and almond during fruit development

At the early stage (T1), both species showed enrichment in cell division and photosynthesis-related processes (Fig. 5). These findings support the role of cell division as a major driver of early fruit size increase, preceding the cell expansion that occurs later in development [10, 12, 61]. Photosynthesis-related processes were also enriched early in development, although the almond mesocarp remained green until splitting. In tomato, green fruit photosynthesis supports seed development [62], which may indicate a similar role in almond kernel development. Here we identified six orthologous gene sets enriched in cell division and seven in photosynthesis across both species (Table S17), underscoring their importance in early fruit development for both peach and almond.

### Biological processes upregulated in peach

Our comprehensive transcriptomic study identified peach-specific regulatory pathways activated at early (T1) middle (T2) and fruit maturity (T3) developmental stages. At T1, pathways such *cyanoamino acid metabolism* and *phenylpropanoid biosynthesis* were enriched, while T2 was characterized by auxin signaling and response. At T3, we observed activation of ethylene-responsive genes, NAC transcription factors, and genes involved in cell wall remodeling and metabolic-processes. Additionally, DNA replication genes were expressed in the early-ripening cultivar ‘Earlygold’.

KEGG enrichment analysis identified three β-glucosidases and one mandelonitrile lyase (MDL) exclusively at T1 in peach type fruits (both in Earlygold and in F1 hybrid), linked to *cyanoamino acid metabolism*. These genes are involved in the synthesis and degradation of prunasin and amygdalin (two common cyanogenic compounds typically found in seeds and teguments), whose breakdown produces benzaldehyde, a volatile abundant in peach mesocarp and declining during fruit development [15, 63–65]. Notably, these genes were not transcribed in almond mesocarp, indicating peach-specific expression. Furthermore, genes associated with the phenylpropanoid pathway and lignin, were significantly expressed at T1 in ‘Earlygold’, consistent with previous reports of pit hardening at the end of S2 stages [66]. Peach-specific softening is driven by *PpPG21* and *PpPG22* [67, 68], which were highly induced after T2 in peach-type fruits but absent in almond. The almond ortholog of *PpPG21* (TexasF1_16883) showed minimal expression at T3, potentially related to tissue softening during mesocarp splitting (Fig. S6).

### Peach upregulated auxin signaling- and auxin-responsive genes, since the middle point of development might explain the differences in fruit development between peach and almond

Auxin strongly influenced peach development transcriptome. At T2, auxin signaling, transport, and response pathways were enriched, with ARF, AUX/IAA, SAUR, and PIN families upregulated. High AUX/IAA expression is typically observed during the late stages of both early- and late-maturing peach cultivars [18, 33]. Among the ripening marker genes identified in the peach–almond DEA, we found two AUX/IAA members (*PpIAA17* and *PpIAA5*), and one *PpGH3*.*1* gene, all upregulated during late peach development (Fig. S6). Their almond orthologs showed no detectable expression. *PpIAA17* is known to be highly expressed during the final ripening stage in both early- and late-ripening peach cultivars [33], while *PpIAA5* has been reported as a marker distinguishing normal from slow ripening (SR) peaches [61]. These findings suggest that these three auxin-related genes are key contributors to peach-specific fruit development and ripening and could serve as genetic markers for these processes in *Prunus spp*. Additionally, “response to auxin” was enriched only in peach at T2 and T3 (Fig. 7), particularly among SAUR genes. Although SAUR genes are regulated by ARFs [69], their functional roles remain unclear. In *Arabidopsis*, SAUR genes act as positive regulators of cell expansion and leaf senescence [70, 71], while in rice, they can negatively regulate auxin synthesis and transport [72]. In apple, auxin is a primary determinant of fruit size [73], and in peach, exogenous auxin treatment increases IAA content and upregulates auxin biosynthetic and SAUR-responsive genes, promoting fruit growth [34]. Here their weak expression detected in almond might suggest partial activation of auxin signaling, which we hypothesize to be linked with the limited mesocarp expansion. Other auxin-induced genes were also active in almond mesocarp. *PpARF6*, which promotes ethylene biosynthesis via *PpACS1* and *PpACO1* [74], was upregulated at T2 in peach, with its almond ortholog showing similar expression pattern. In tomato, *SlARF6* enhances sugar and chlorophyll yet inhibits ripening [75, 76], while apple and cherry homologs (*MdARF13*, *PavARF8*) repress anthocyanin and cell-wall softening genes [77, 78]. These results suggest that auxin-regulated genes start diverging at T2, with almond maintaining expression but likely differing in regulatory outcome.

### DNA replication genes are associated with fruit expansion in the early ripening cultivar Earlygold

At T3, ‘Earlygold’ activated “DNA replication” and endoreduplication genes (MCMs, WEE1, CCS52A1), linking cell enlargement to final fruit size. These genes are associated with cell enlargement and fruit size linking late-stage fruit expansion to endoreduplication, in fleshy fruits like in tomato [13, 79–82]. Both the cell number and cell enlargement contribute to the final peach fruit size, with the cell number accounting for approximately 60% of the total size [10, 83]. Previous findings showing high endoreduplication gene expression also in late-ripening peach cultivars, which contributed to bigger fruits than the SR mutant [61]. The absence of replication gene activation in almond at T3 (Fig. 7 and Fig. S7) supports endoreduplication’s role in driving mesocarp´s expansion variation among peaches and between peach and almond.

### Biological processes upregulated in almond

#### Upregulated genes in almond during fruit development are related to secondary metabolism and hormone signaling linked to defense responses

Almond exhibited the highest number of DEGs between T1 and T2, with only minor transcriptomic changes between T2 and T3. At T1, “metal ion binding” and at commonly at T2 and T3 “monooxygenase activity” were enriched through CYP450s and terpene synthases, enzymes central to amygdalin and volatile biosynthesis [15, 63, 84]. At T2, almond DEGs were enriched in *biosynthesis of various plant secondary metabolites*, including saponin adjuvants, coumarin biosynthesis (beta-amyrins, β-glucosidases), and a polyphenol oxidase, absent in peach-type fruits. However, in peach, PPO activity has been reported during early fruit development [85]. Additionally, 2,957 genes unmapped to peach via *liftoff* were considered almond-specific (Fig. 6), with 141 and 43 differentially expressed between T1 vs. T2 and T2 vs. T3, respectively. At T2, GO enrichment identified six WAK genes, which are known to link the cell wall to the plasma membrane and facilitate intracellular signaling [86]. WAK-like kinases (WAKLs) are key components of innate immune responses in species such as *Arabidopsis*, maize, and orange [87–90]. Also at T2, almond-specific terpene biosynthetic genes (alpha-pinene and alpha-phellandrene synthases) were expressed, consistent with volatile accumulation [84]. Overall, T2 almond gene expression predominantly reflects secondary metabolism and immune responses, in contrast to the hormone signaling pathways that drive fruit development and ripening in peach at the same stage.

At T2, almond upregulated genes were enriched for terms related to both *abscisic* and *ethylene activated signaling pathway*, as well as *defense response* (Fig. 5). DEGs annotated with *abscicic acid-activated signaling pathway* also shared exclusive GO terms such as *protein phosphatase inhibitor activity*, *signaling receptor activity* and *defense response* (Fig. S2 F). These findings suggest that ABA signaling in almond at T2 primarily activates defense-related genes, rather than coordinating fruit ripening, as observed in non-climacteric fruits [17]. Major latex proteins (MLP), which are commonly associated with abiotic and biotic stress responses in species, such as sugar beet (*Beta vulgaris*), tobacco (*Nicotiana benthamiana*) and grape vine [91–93], were also detected. Nevertheless, in kiwifruit, MLP was the first ripening-related protein characterized, and in apple, MLPs and PRs are induced during fruit ripening [94, 95]. We also identified four AP2/ERF genes, one ERF98-like gene (TexasF1_G29703) and three ERF1b genes (TexasF1_G23803, TexasF1_G899, TexasF1_G29702). The peach orthologs Pp*ERF98*-1,2 have been reported as negative regulators of peach gummosis disease, acting through the induction of *PpERF1* and activation of the ERF branch in JA/ET signaling pathway, while suppressing the SA-dependent defense pathway [96]. These ERF genes are induced by both pathogen treatment and ethylene, suggesting a role in stress response. This is further supported by the expression of the *PdACS1* (TexasF1_G8820), which showed a similar expression pattern to peach at T2 (Fig. 8). This suggests that ethylene in almond may be involved in regulating biotic and abiotic stress responsive genes, rather than promoting ripening [97–99].

#### The ortholog of transcription factor PpNAC5 is expressed in the almond mesocarp during fruit development, suggesting that it does not function as a ripening regulator, as in peach fruit

GSEA revealed that *regulation of DNA-templated transcription* was specifically enriched among genes upregulated in almond at T2 and T3 compared to peach at the respective stages (Fig. 7, Table S11-12). Notably, *PpNAC5* was upregulated during peach development (T1 vs. T2) and in almond compared to peach at T2 and T3. *PpNAC5* is a candidate gene for determining maturity date in peach [60, 100], and a major QTL for maturity date has been mapped to chromosome G4 in sweet cherry (*Prunus avium*) and *Prunus cerasus* [101, 102]. A deletion at the *PpNAC5* gene resulting in its loss of expression has been linked to the slow ripening phenotype in peach, in which fruit ceases growth and ripening at the S3 stage [32]. Adjacent transcription factors *PpNAC5* and *PpNAC1* have been shown to co-regulate peach ripening genes [23]. For instance, *PpBL*, a key gene for the blood flesh trait in peach, forms heterodimers with *PpNAC1* to activate *PpMYB10*.*1* transcription during late ripening [103]. In middle-maturity date peach cultivars, *PpNAC1* expression increases throughout development, peaking at ripening, while *PpNAC5* shows minimal expression at this [23]. Our transcriptomic data, validated by qPCR, confirmed these patterns in peach and revealed constitutive but lower expression of the almond *PdNAC1* ortholog, alongside higher expression of the almond *PdNAC5* ortholog (Fig. 8). This suggests possible species-specific divergence or blocking downstream the ripening process in almond. Structural variation or polymorphisms may impact the function of these NAC-TFs. The *Alf* gene in the TxE interspecific population maps at G4, encompassing both NAC-TFs, leading to the almond-type fruit phenotype, characterized by a lack of mesocarp expansion and ripening ability [7]. Moreover, DNA methylation of both NACs has been linked to the early-ripening phenotype in a peach sport mutant [104]. These findings suggest that polymorphisms or epigenetic modifications may underlie the non-ripening mesocarp phenotype in almond, despite detectable NAC expression.

The TxE interspecific populations enabled mapping of the two major genes distinguishing almond from peach fruit, including *Alf* and *Jui* [7, 8]. Our data provides evidence for the two NAC TF at the *Alf* candidate region, however *Jui* candidate region remains extensive and was not explored here. Future directions will involve fine mapping of *Alf*,* Jui* and *DBF2* genes and integration of the present data with fruit development transcriptomic profiles of introgression lines, to provide strong evidence for the causal genes.

### Identification of ASE in the interspecific F1 hybrid between peach and almond during fruit development

ASE is an important regulatory mechanism that recently helped to identify a peach gene conferring aphid resistance [105]. In this study, we investigated ASE during F1 hybrid fruit development using the peach genome annotation due to its higher BUSCO completeness (Fig. S5). Our analysis focused on genes containing heterozygous SNPs within coding sequences.

Although ASE detection methodologies have improved in accuracy [106], challenges remain, particularly in reliably distinguishing maternal and paternal alleles and establishing robust thresholds for ASE detection. Applying conservative criteria, we analyzed 22,118 genes (representing 82.3% of whole peach annotation). Under these stringent conditions, fewer than 1% of genes showed clear ASE at each developmental stage: 79 peach-ASE genes and 27 almond-ASE genes at T1; 99 peach-biased and 51 almond-biased at T2; and 119 peach-biased and 77 almond-biased at T3. While higher ASE levels have been reported in pear [107] and apple [44], those studies used less stringent thresholds. Our results are consistent with previous findings of limited transcriptional and epigenetic changes in this F1 hybrid [30] and confirm that ASE detection is highly sensitive to threshold selection.

ASE patterns varied across genes and developmental stages. Most ASE genes reflect parental expression patterns, with intermediate expression levels in the hybrid and few cases of positive or negative transgression expression. This aligns with previous reports showing additive expression effects in F1 hybrids [41, 43]. Notably, *PpIAA5*, exhibited peach-biased ASE exclusively at these stages. We also observed developmental stage-dependent ASE patterns. For example, a *PMEI* gene showed peach-biased ASE at T1 and almond-biased ASE at T3 (Fig. S9B). *PME* and *PMEI* genes are involved in pectin degradation and are known to peak in melting flesh peaches (MF) [108]. Their almond-biased ASE may contribute to the F1 hybrid slower mesocarp softening compared to the MF cultivar ‘Earlygold’.

At T3, peach-biased ASE genes included those involved in carotenoid biosynthesis (*PpCCD4*, *PpNCED3*) and lipoxygenase activity (*PpLOX3*). *PpCCD4* regulates peach flesh color [109], while *PpNCED3* contributes to ABA biosynthesis [110]. LOXs are known to regulate plant development, fruit ripening, and stress responses [111–114]. In peach, ethylene-dependent LOXs modulate aroma volatile production during ripening [115, 116], and in apricot, *LOX2* serves as a ripening marker [117]. We hypothesize that the peach-specific ASE of ripening-related genes *PpIAA5*, *PpCCD4*, and *PpLOX3*, which are uniquely expressed in the peach parent, contributes to the F1 hybrid’s peach-like ripening phenotype. Interestingly, *PpNCED3* showed peach-biased ASE despite detectable expression in almond. As a key gene in ABA biosynthesis during late ripening [110], *PpNCED3* is regulated by *PpERF3*, which links ethylene and ABA signaling [118]. We identified a 14 bp deletion in the ERF-binding promoter region of the almond ortholog, which may explain the preferential peach-biased ASE of *PpNCED3*. ASE in F1 hybrids often results from cis-regulatory divergence, including transposable element insertions and high SNP density in promoter regions [41, 44, 45]. Our findings confirm ASE in the F1 hybrid during fruit development, suggesting that cis-regulatory differences between parental genomes may underlie these patterns.

## Conclusions

This comparative transcriptomic study provides new insights into the transcriptional changes shaping fruit development and ripening across *Prunus* species. Early fruit growth in both peach and almond is driven by conserved cell division and photosynthetic processes, their transcriptomic trajectories diverge sharply from mid-development onwards. Peach-type fruits exhibited a marked activation of auxin signaling, DNA replication, and ethylene-mediated ripening pathways, supported by the upregulation of genes such as *PpIAA5*, *PpIAA17*, *PpPG21/22*, and *PpNAC1/5*. In contrast, almond maintained a relatively stable transcriptome dominated by secondary metabolism, and abscisic acid- and ethylene-related stress response genes. Allele-specific expression analysis in the F1 hybrid uncovered stage-dependent parental biases, notably peach-biased expression in ripening-associated genes (*PpCCD4*, *PpNCED3*, *PpLOX3*), likely driven by cis-regulatory divergence. These findings enhance the understanding of genetic regulation in fruit development within *Prunus spp* and would help to identify candidate genes for important traits related to fruit development identified in interspecific populations between peach and almond such as *DBF2*, *Jui*, and *Alf.*

## Supplementary Information

Supplementary Material 1.

Supplementary Material 2.

## Data Availability

The raw data generated and analyzed during the current study is available in the European Nucleotide Archive (ENA) repository under the project number PRJEB94283.
