# Supplementary material for: Comparative transcriptomics among peach, almond and their interspecific F1 hybrid reveal key common and species-specific regulatory pathways involved in fruit development
Source: BMC Plant Biol. 2026 May 12;26:1135. doi: 10.1186/s12870-026-08863-6 (PMC13335376; doi:10.1186/s12870-026-08863-6)
Supplement: Supplementary file 1 — Supplementary Material 1. [file 12870_2026_8863_MOESM1_ESM.pdf]

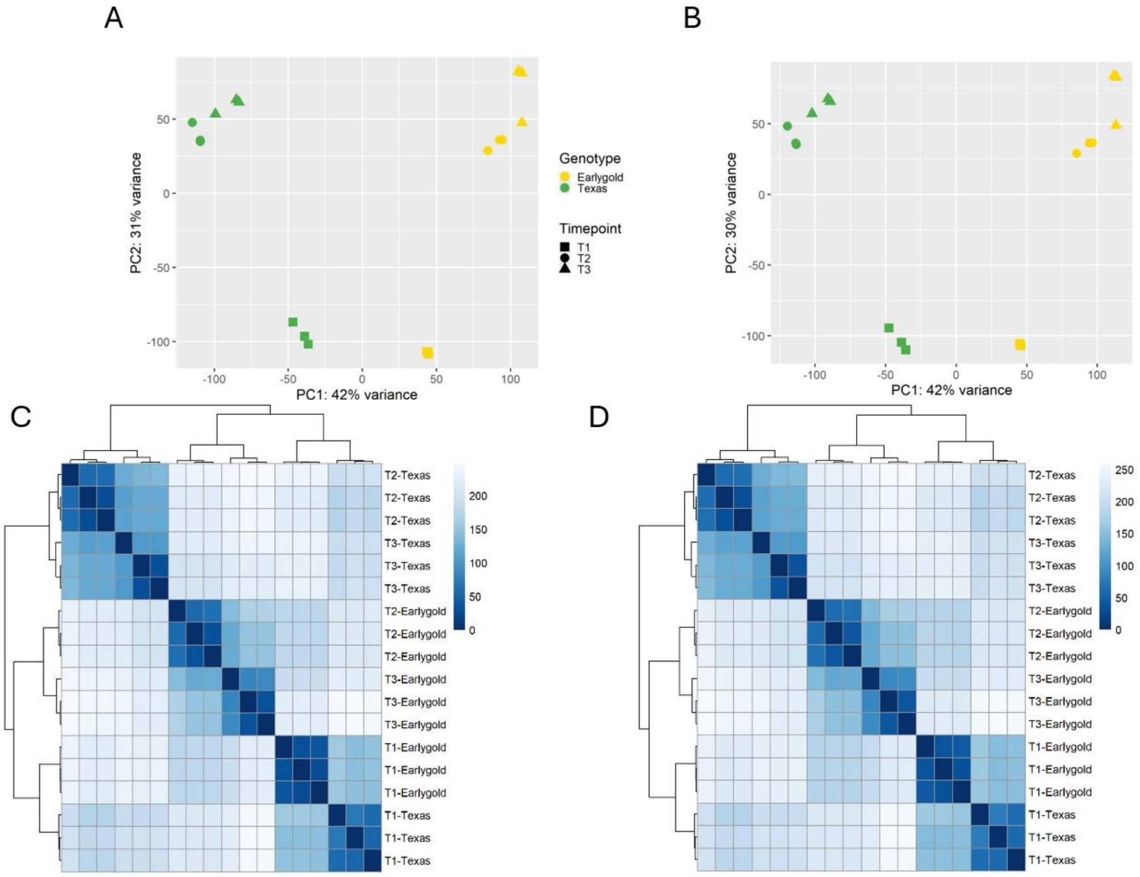

Supplementary Figure 1. Exploratory analysis and visualization of r-log transformed expression data with dataset including only 'Earlygold' and 'Texas' samples. The filtered/normalized data used for exploratory analysis were obtained from reads mapped to Lovell v2.0 (A, B) and Texas v3.0 Phase 1 (P1) (C, D) reference genomes. A sample-to-sample Euclidean distance matrix heatmap (A,C) shows the overall similarity between samples, and a Principal Component Analysis (PCA) plot (B,D) is projected onto the 2D plane of the two principal components (PC).

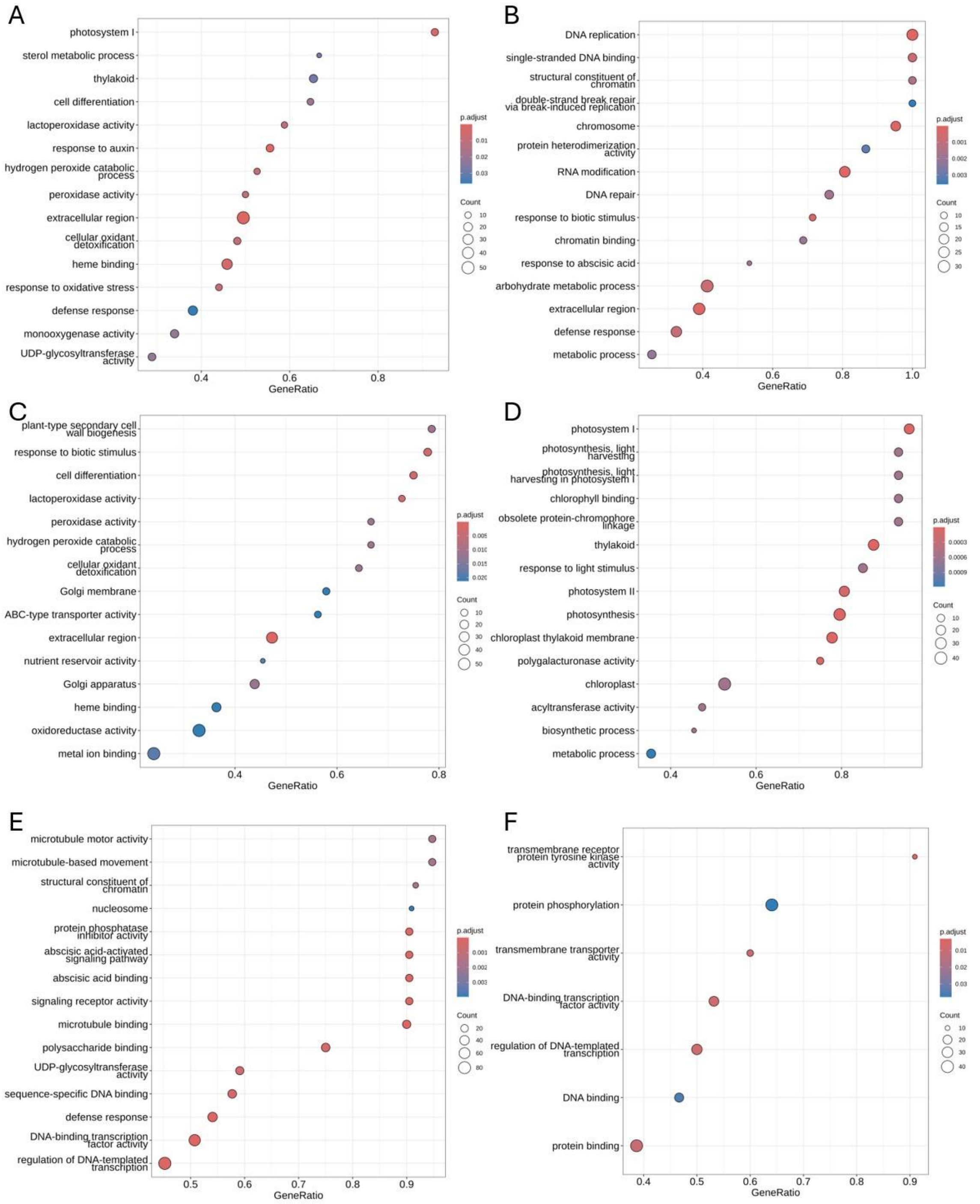

Supplementary Figure 2. Dot plots of the top GO terms enriched at the GSEA analysis of DEGs in T1 vs T2 (left) and in T2 vs T3 (right) in 'Earlygold' (A & B), F1 hybrid (C & D) and 'Texas' (E & F). Top 15 GO terms enriched are plotted.

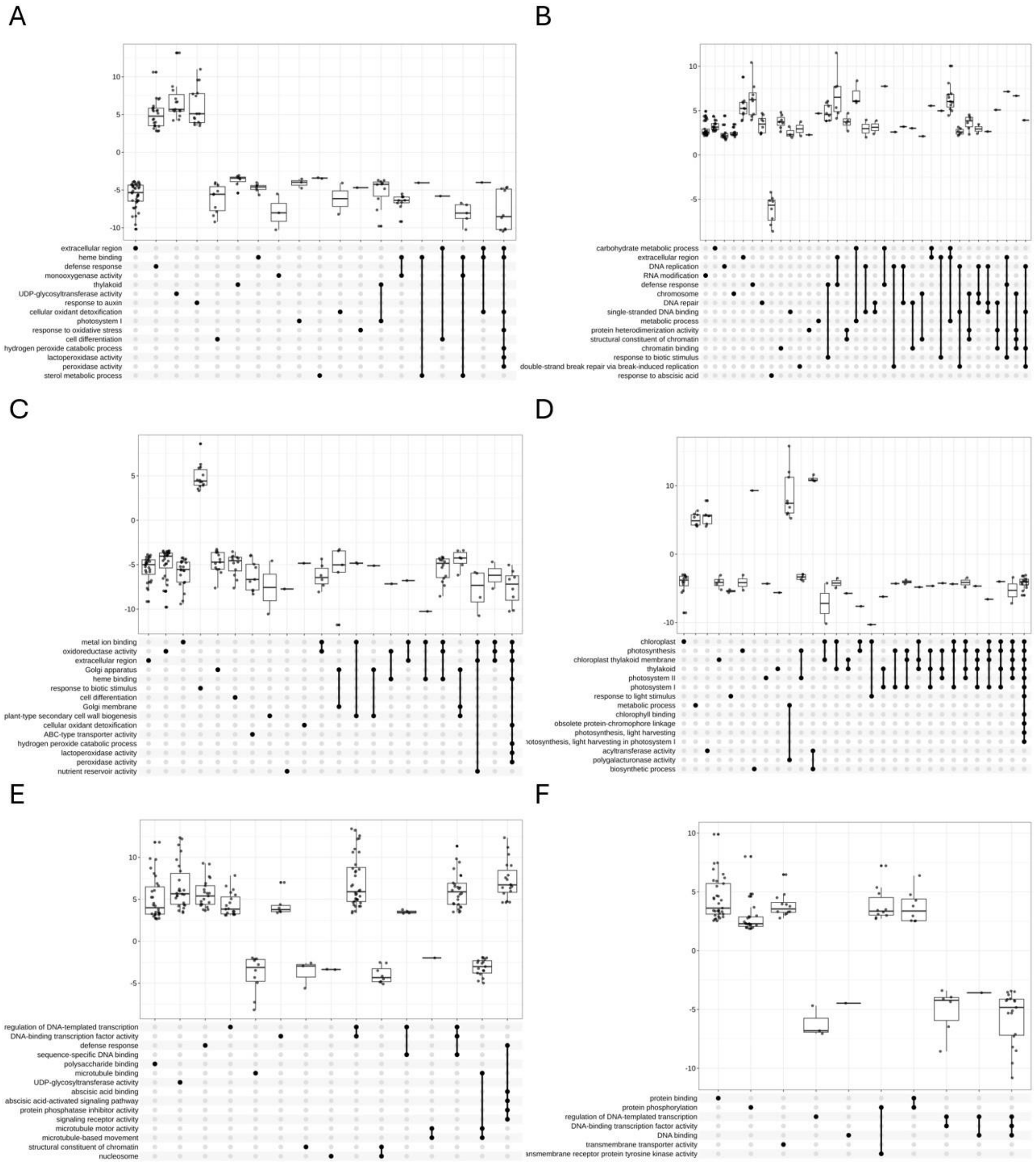

Supplementary Figure 3. Upset plots of the top GO terms enriched at the GSEA analysis of DEGs in T1 vs T2 (left) and in T2 vs T3 (right) of fruit development in 'Earlygold' (A & B), F1 hybrid (C & D) and 'Texas' (E & F). In y-axis logFC at T2 compared to T1 and at T3 compared to T2, for the first and second half of fruit development respectively, is plotted. Dots at the graph represent genes annotated with the corresponding GO terms. Unique dots at the x-axis show unique terms for the annotated genes multiple dots linked with solid lines show shared GO terms for the annotated genes.

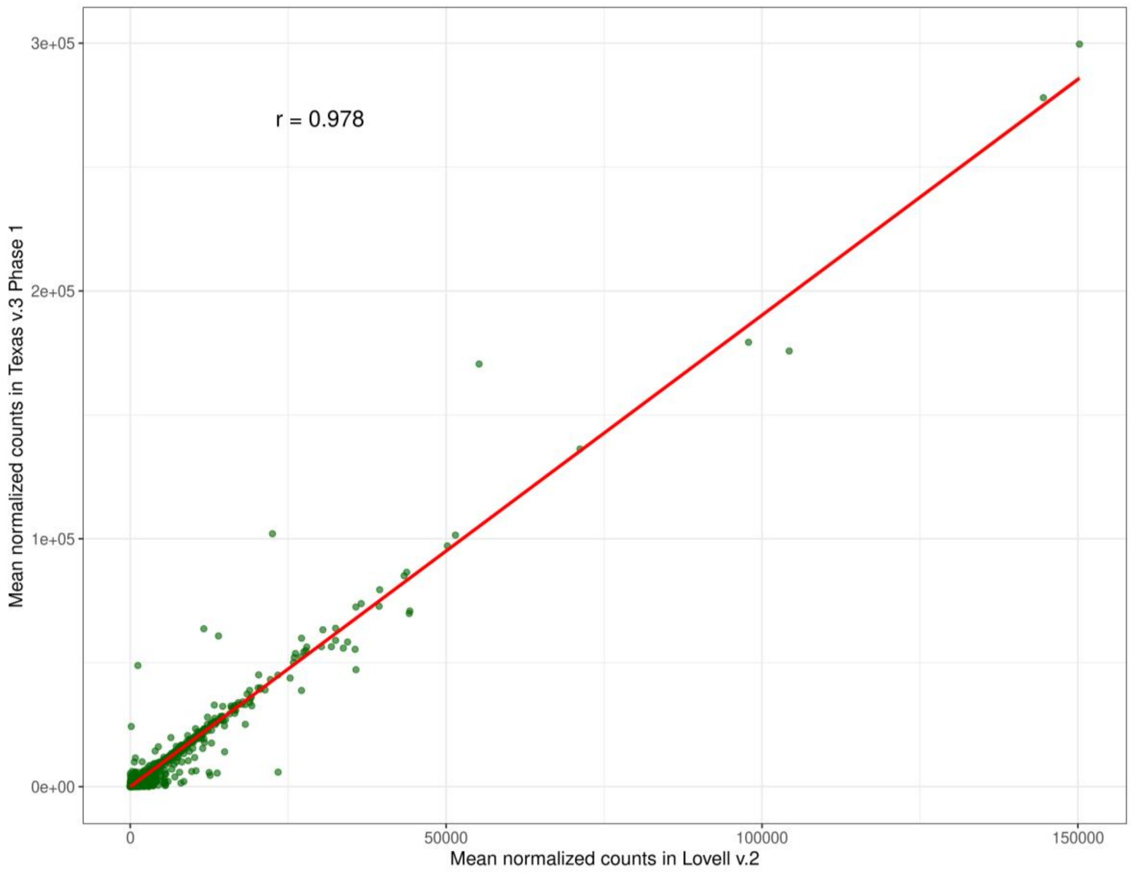

Supplementary Figure 4. Pearson correlation of mean normalized gene counts for all samples using peach (Lovell v2.1) reference and almond (Texas v.3) reference genomes. Green dots represent the orthologous genes expressed in all 'Earlygold' and 'Texas' samples, the red line represents the regression line using linear regression model and r as the correlation coefficient of the model.

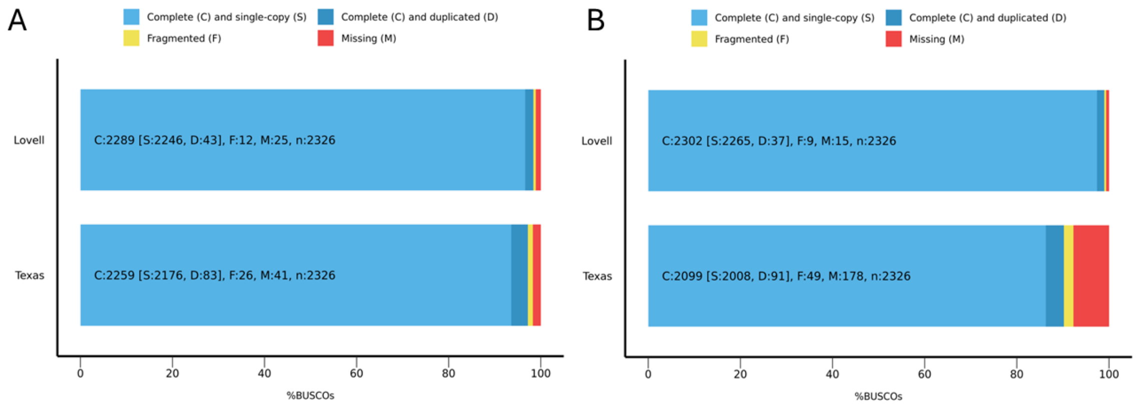

Supplementary Figure 5. Busco assessments results for reference A) genomes and B) protein annotations. Peach reference genome (Lovell v2.1)has less missing and duplicated genes than Texas v.3 of the BUSCO analysis, indicating that is the most suitable genome to transfer their annotated genes by liftoff. In addition, peach annotated genes show that they are of better quality to use for the correspondence and the DEA analysis.

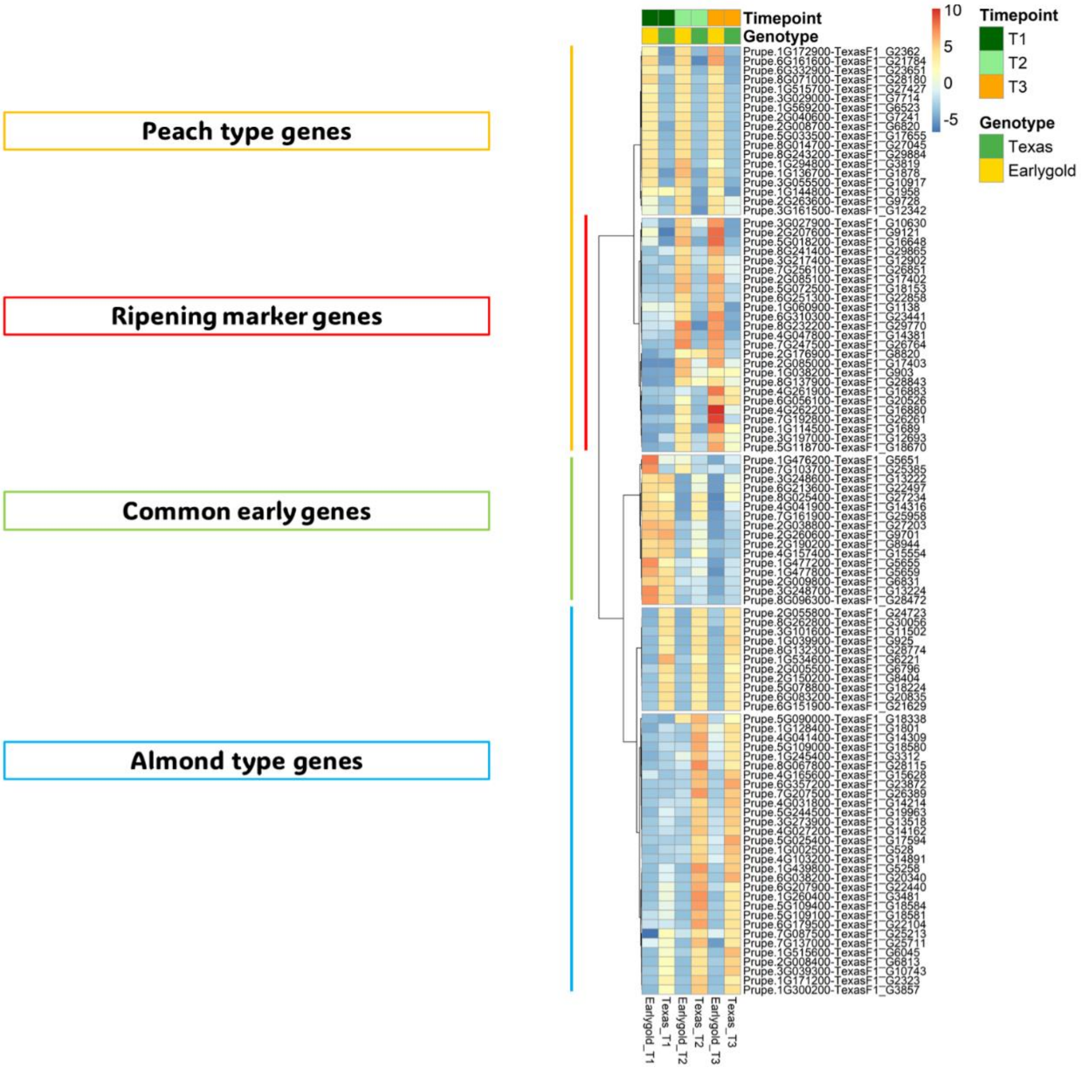

Supplementary Figure 6. Expression heatmap of r-log transformed data for the 100 most variable genes in the orthologous gene dataset. Colored boxes represent mean gene expression of three biological replicates for each genotype and timepoint. Genotype and timepoint information are shown with colored boxes and r-log expression value with a colored bar at the top-right. Genes hierarchical clustering is represented as a dendrogram on the left. An r-log count matrix clustering was set with a cut-tree cut-off of 5.

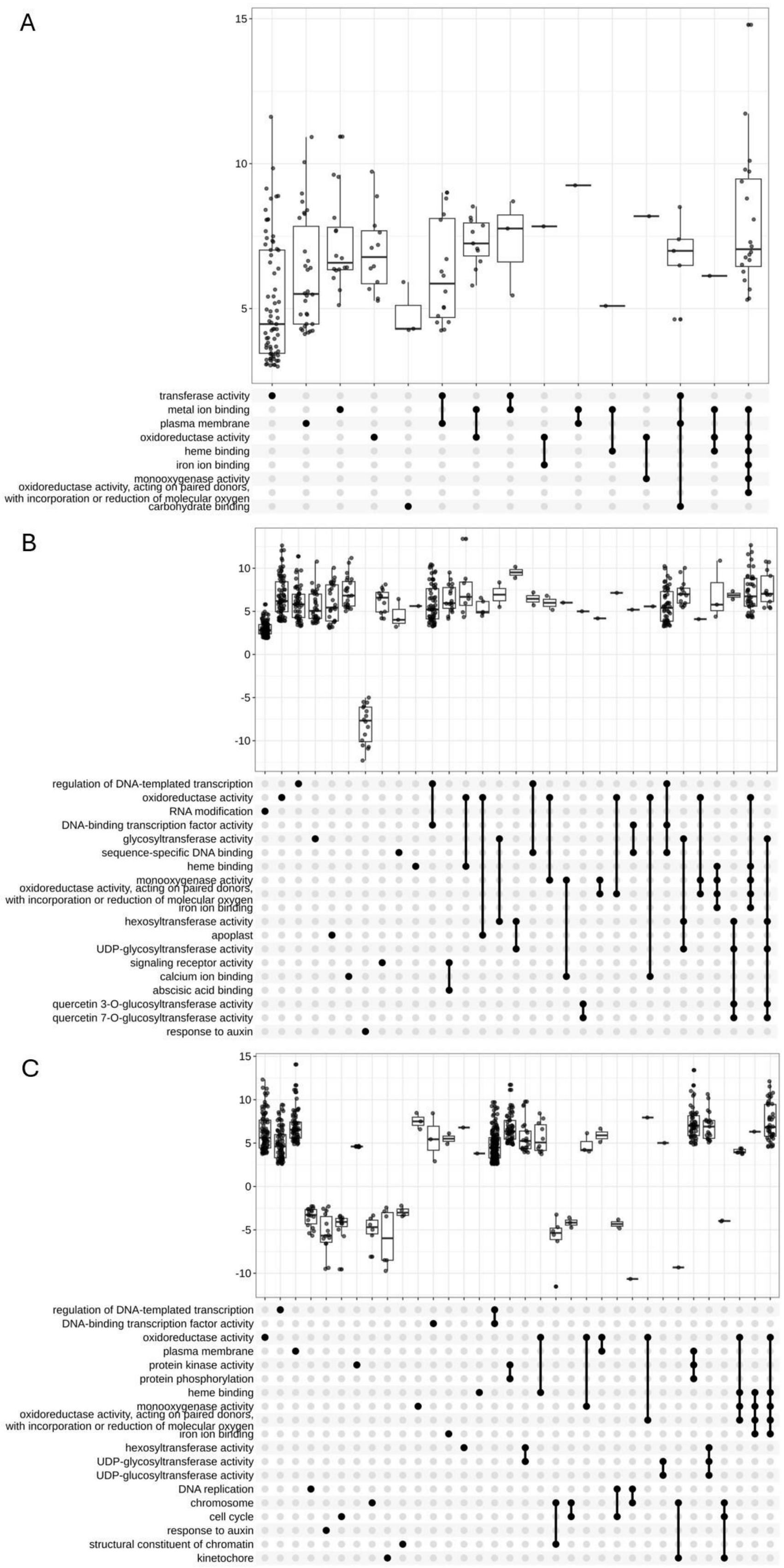

Supplementary Figure 7. Upset plots of the top GO terms enriched at the GSEA analysis for DEGs between 'Earlygold' and 'Texas' at (A) T1 (B) T2 and (C) T3. In y-axis logFCs of 'Texas' compared to 'Earlygold' genes are plotted. Dots at the graph represent genes annotated with the corresponding GO terms. Unique dots at the x-axis show unique terms

for the annotated genes multiple dots linked with solid lines show shared GO terms for the annotated genes.

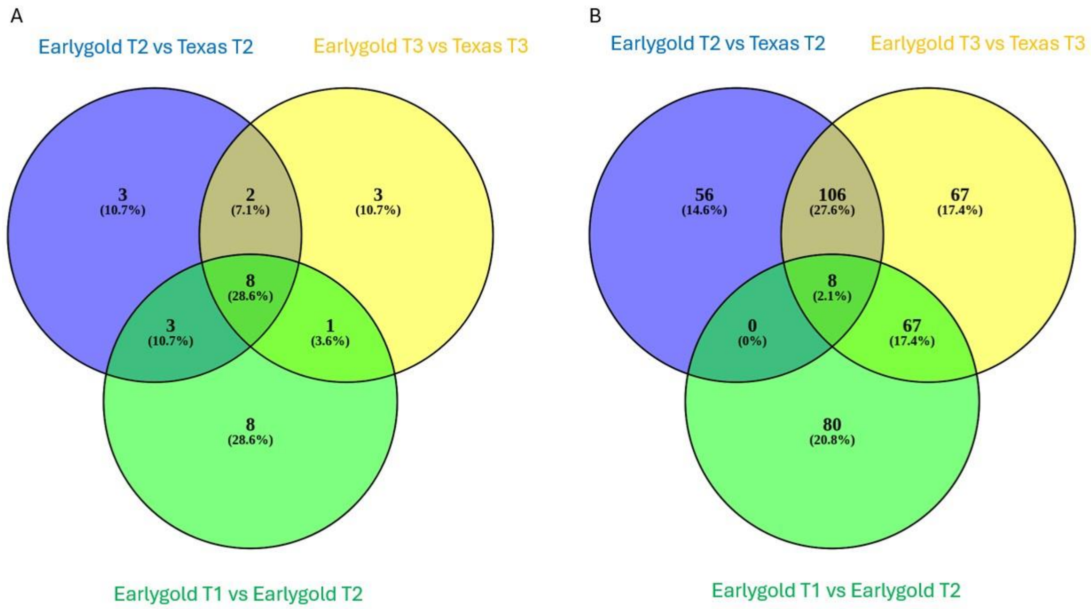

Supplementary Figure 8. Venn diagrams of total number of genes enriched in A) “response to auxin” and B) “regulation of DNA-templated transcription” found in the three comparisons, within peach development and between peach - almond development.

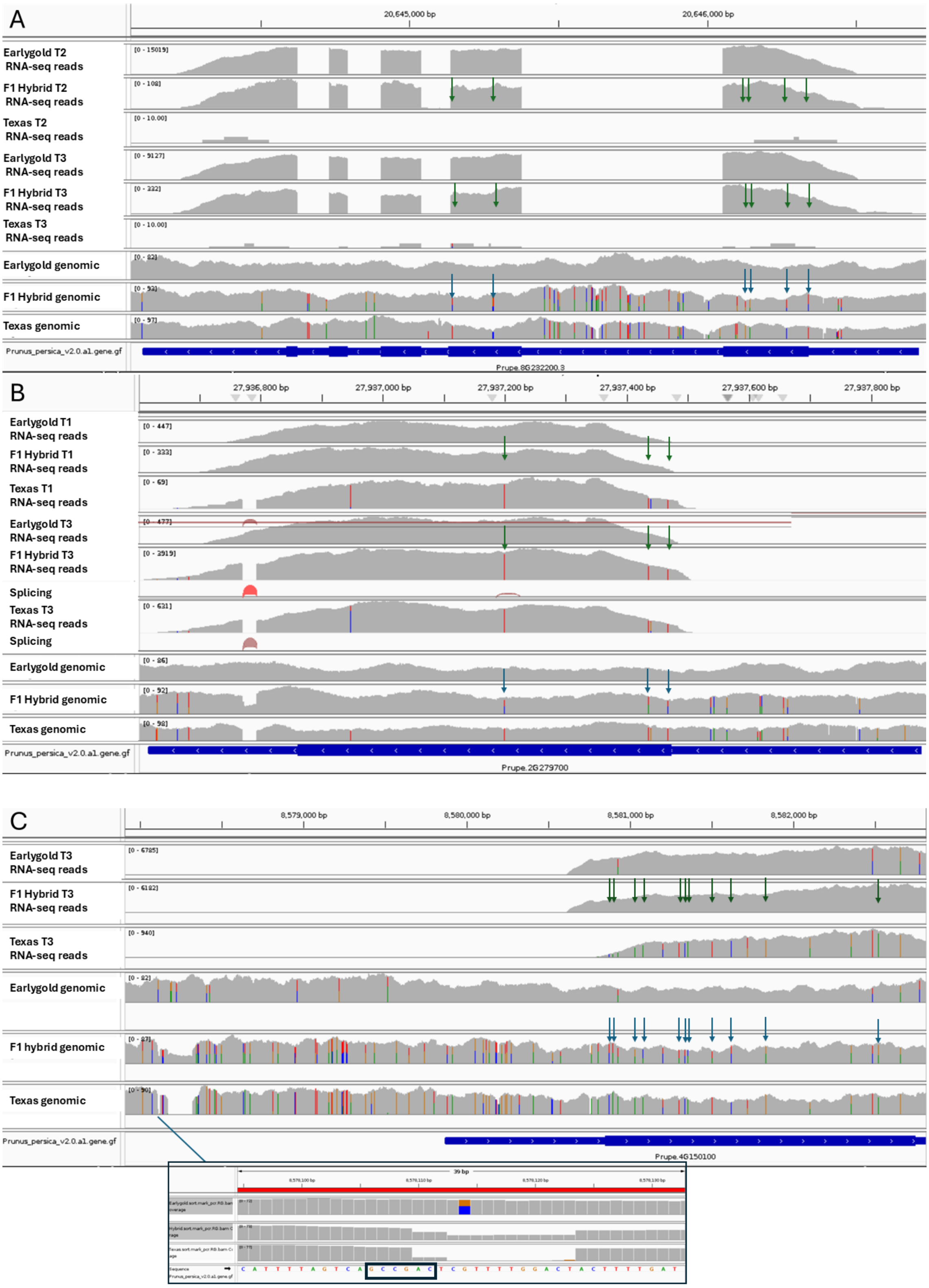

Supplementary Figure 9. IGV screenshots of RNA-seq reads and genomic DNA reads for visualization of ASE in F1 hybrid fruit samples. Grey bars show read coverage and coverage range is shown for each track inside the brackets. Blue arrows show the heterozygous SNPs at the genomic reads of the F1 hybrid, used to distinguish the two parental alleles. Green arrows show the corresponding positions at the RNA-seq reads of F1 hybrid with ASE. A) Example of ASE following parentals intermediate expression of *PpIAA5* (Prupe.8G232200)

at T2 and T3, with high expression in peach and no expression in almond. B) Example of dual ASE behavior of a PMEI (Prupe.2G279700), with peach ASE at T1 and an almond ASE at T3, with a splicing site change. C) Example of peach ASE for *PpNCED3* (Prupe.4G150100) with gene expression in both parental fruits at T3. An 14bp deletion disrupting the ERF binding box ‘GCCGAC’ at the *PpNCED3* promoter region (-2740bp from ATG), is shown in the zoom-in box, with ‘Texas’ homozygous and F1 hybrid heterozygous for the deletion.
